# Female glucagon receptor knockout mice are prone to steatosis but resistant to weight gain when fed a MASH‐promoting GAN diet and a high‐fat diet

**DOI:** 10.14814/phy2.70235

**Published:** 2025-02-21

**Authors:** Katrine D. Galsgaard, Emilie Elmelund, Jenna E. Hunt, Mark M. Smits, Trisha J. Grevengoed, Christina Christoffersen, Nils J. Færgeman, Jesper Havelund, Nicolai J. Wewer Albrechtsen, Jens J. Holst

**Affiliations:** ^1^ Department of Biomedical Sciences, Faculty of Health and Medical Sciences University of Copenhagen Copenhagen Denmark; ^2^ Novo Nordisk Foundation Center for Basic Metabolic Research, Faculty of Health and Medical Sciences University of Copenhagen Copenhagen Denmark; ^3^ Department of Internal Medicine Radboud University Medical Center Nijmegen The Netherlands; ^4^ Department of Clinical Biochemistry Rigshospitalet, University of Copenhagen Copenhagen Denmark; ^5^ Department of Biochemistry and Molecular Biology University of Southern Denmark Odense Denmark; ^6^ Department of Clinical Biochemistry Copenhagen University Hospital – Bispebjerg Copenhagen Denmark; ^7^ Department of Clinical Medicine, Faculty of Health and Medical Sciences University of Copenhagen Copenhagen Denmark

**Keywords:** cholesterol, glucagon, high‐fat diet, steatosis, triglycerides

## Abstract

Glucagon is secreted from the pancreatic alpha cells and regulates not only hepatic glucose production, but also hepatic lipid and amino acid metabolism. Thus, glucagon provides a switch from hepatic glucose and lipid storage towards lipid and amino acid breakdown fueling glucose production during fasting. However, the effects of genetic deletion of the glucagon receptor on lipid metabolism are unclear. We therefore assessed parameters of lipid metabolism in fasted and non‐fasted male and female mice with permanent whole‐body deletion of the glucagon receptor (*Gcgr* ^−/−^ mice). To investigate whether *Gcgr* ^−/−^ mice tolerated a diet promoting metabolic dysfunction‐associated steatohepatitis (MASH) and steatosis, we fed female *Gcgr* ^−/−^ mice the Gubra Amylin Nonalcoholic steatohepatitis (GAN) diet and high‐fat diet (HFD), respectively. We found that non‐fasted *Gcgr* ^−/−^ mice fed standard chow showed hypercholesterolemia and increased liver fat (borderline significant in non‐fasted male *Gcgr* ^−/−^ mice, but significant in the remaining groups). In the fasted state these changes were insignificant due to fasting‐induced steatosis. When challenged with a GAN diet and HFD, female *Gcgr* ^−/−^ mice were prone to steatosis and dyslipidemia but resistant to weight gain. Taken together, our data highlight glucagon as an important physiological regulator of not just glucose, but also hepatic lipid metabolism.

## Graphical abstract

Female glucagon receptor knockout mice are prone to steatosis when fed a MASH‐promoting GAN diet and a high fat diet.

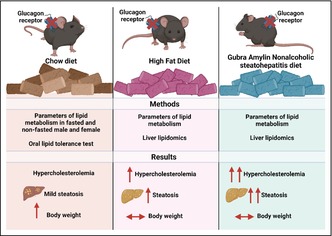

## 1 INTRODUCTION

During conditions of decreasing blood glucose levels, glucagon is secreted from the pancreatic alpha cells. Upon reaching its primary target organ, the liver, glucagon increases not only hepatic glucose production (Jiang & Zhang, 2003; Ramnanan et al., 2011), but also amino acid metabolism (ureagenesis) (Fitzpatrick et al., 1977; Hamberg & Vilstrup, 1994), and lipid oxidation (Longuet et al., 2008; Pegorier et al., 1989; Roden et al., 2000; Witters & Trasko, 1979). Thus, glucagon increases plasma glucose concentrations (Jiang & Zhang, 2003; Ramnanan et al., 2011), while lowering concentrations of plasma amino acids (Boden et al., 1984; Fitzpatrick et al., 1977) and hepatic triglycerides (TG) (Jiang & Zhang, 2003; Roden et al., 2000; Witters & Trasko, 1979). Moreover, by decreasing very low‐density lipoprotein (VLDL) secretion from the liver (Longuet et al., 2008; Pegorier et al., 1989) glucagon reduces plasma TG and cholesterol concentrations (Amatuzio et al., 1962; Eaton, 1973; Guettet et al., 1988; Guettet, Rostaqui, Mathe, et al., 1991; Guettet, Rostaqui, Navarro, et al., 1991; Rudling & Angelin, 1993). These regulatory effects of glucagon are now often referred to as the liver‐alpha cell axis (Richter et al., 2022).

Subjects with metabolic dysfunction‐associated steatotic liver disease (MASLD) show increased plasma concentrations of glucagon and amino acids (Junker, 2017; Junker et al., 2016; Wewer Albrechtsen et al., 2018) suggesting that a disruption of the liver‐alpha cell axis has occurred. Supporting this, in both mice (Winther‐Sørensen et al., 2020) and humans (Heebøll et al., 2022; Suppli et al., 2020), steatosis has been linked to hepatic glucagon resistance which in turn affects both amino acid and lipid metabolism. The effect on amino acid metabolism shows up as a reduced glucagon‐induced decrease in plasma amino acid concentrations (Suppli et al., 2020) and a reduced increase in ureagenesis (Winther‐Sørensen et al., 2020), while the effect on lipid metabolism is evident as a reduced glucagon‐induced suppression of hepatic VLDL secretion (Heebøll et al., 2022). In the presence of a fatty liver, glucagon resistance may therefore create a vicious cycle of hyperglucagonemia, hyperaminoacidemia, hepatic lipid accumulation, and dyslipidemia (Galsgaard, 2020).

The glucagon receptor has been shown to be required for control of lipid metabolism during the adaptive metabolic response to fasting, as hepatic lipid oxidation failed to rise in response to fasting in mice with permanent deletion of the glucagon receptor (*Gcgr* ^−/−^ mice) causing increased hepatic TG secretion and increased plasma TG concentrations (Longuet et al., 2008). However, the effects of genetic deletion of the glucagon receptor on plasma and liver TG concentrations remain controversial (Conarello et al., 2007; Gelling et al., 2003; Parker et al., 2002; Sinclair et al., 2008). To investigate whether the inconsistent findings might be due to differences in feeding‐fasting‐regimes and perhaps the sex of the mice, we assessed lipid metabolism in male and female *Gcgr* ^−/−^ mice in the non‐fasted as well as the fasted state. We also investigated how female *Gcgr* ^−/−^ mice respond to a metabolic dysfunction‐associated steatohepatitis (MASH)‐promoting diet as well as a high‐fat diet (HFD; promoting steatosis).

This disentangling of glucagon's role in lipid metabolism is highly important considering the current development of glucagon‐based drugs to treat obesity and MASLD (Coskun et al., 2022; Day et al., 2009; Finan et al., 2015; Henderson et al., 2016; Urva et al., 2022). Our results suggest that permanent genetic deletion of the glucagon receptor may result in steatosis and hypercholesterolemia and thus highlight glucagon as an important physiological regulator of not just glucose, but also hepatic lipid metabolism.

## 2 METHODS

### 2.1 Mouse studies

Animal studies were conducted at the animal facilities at the Faculty of Health and Medical Sciences, University of Copenhagen, Copenhagen, with permission from the Danish Animal Experiments Inspectorate, Ministry of Environment and Food of Denmark, permit 2018‐15‐0201‐01397. All studies were approved by the local ethical committee; Department of Experimental Medicine – University of Copenhagen. Glucagon receptor knockout (*Gcgr* ^−/−^) mice (C57BL/6J^Gcgrtm1Mjch^) and their wild‐type littermates (*Gcgr* ^+/+^ mice) were bred in‐house with permission from Dr. Maureen J. Charron as described previously (Gelling et al., 2003). Female mice were housed in groups of four to eight and male mice in groups of two to six in individually ventilated cages. All mice followed a light cycle of 12 h (lights on 6 am to 6 pm) and were allowed a minimum of 1 week of acclimatization before being included in any experiment.

### 2.2 Characterization of glucagon receptor knockout mice in the non‐fasted and fasted state

Female and male *Gcgr* ^−/−^ and *Gcgr* ^+/+^ mice, 14–18 weeks of age, had ad libitum access to chow (Autoclavable complete breeding vegetal diet for rats, mice and hamsters, SAFE® Complete Care Competence) or were fasted overnight (16 h). Blood glucose concentrations were measured after tail tip puncture using a handheld glucometer (Accu‐Chek® Mobile, catalog no. 05874149001; Roche Diagnostics, Mannheim, Germany), and the mice were weighed. Immediately after, blood was collected from the retrobulbar plexus using ethylenediaminetetraacetic acid (EDTA) coated capillary tubes (Micro Haematocrit Tubes, ref. no. 167313; Vitrex Medical A/S, Herlev, Denmark), subsequently stored on ice until spun (9000 g, 4°C, 10 min) and stored at −80°C. The mice were then euthanized by cervical dislocation and the liver, pancreas, and kidneys were excised, weighed, and snap‐frozen in liquid nitrogen. Finally, the tip of the ear was cut with a scissor washed with ethanol for genotyping.

In a separate experiment, female and male *Gcgr* ^−/−^ and *Gcgr* ^+/+^ mice, 7–16 weeks of age, had ad libitum access to chow. Blood glucose concentrations were measured as described, and the mice were weighed and sedated using ketamine (catalog no. 511485, Ketaminol®, Intervet, Merck & CO., New Jersey, dose 100 mg/kg body weight) in combination with isotonic saline (0.9% NaCl) and xylazine (catalog no. 148999, Rompun® vet, Bayer AG, Leverkusen, Germany, dose 10 mg/kg body weight) given as an intraperitoneal injection. After sedation, blood was collected from the vena cava and stored on ice in EDTA coated Eppendorf tubes until spun (6500 g, 4°C, 10 min). Plasma was collected and stored at −80°C. Tissues were collected as described. To perform fast protein liquid chromatography (FPLC) analysis, aliquots of the plasma collected from each of the *Gcgr* ^−/−^ and *Gcgr* ^+/+^ mice were pooled (according to the groups) in prechilled Eppendorf tubes and stored at −80°C until analysis as described in (Galsgaard et al., 2022).

#### 2.2.1 Oral lipid tolerance test

*Gcgr* ^−/−^ (females *n* = 7 and males *n* = 8) and *Gcgr* ^+/+^ (females *n* = 5 and males *n* = 9) mice, 10–14 weeks of age, were fasted overnight (16 h). The following morning, blood was collected from the retrobulbar plexus and blood glucose concentrations were measured using a handheld glucometer. Immediately after, the mice received 10 μL/g body weight of olive oil and blood was collected 30, 120, and 180 min after lipid administration. The mice were then killed by cervical dislocation and tissue for genotyping was collected as described.

#### 2.2.2 Gubra amylin nonalcoholic steatohepatitis diet and high‐fat diet challenge

Female *Gcgr* ^−/−^ and *Gcgr* ^+/+^ mice, 9–16 weeks of age, had ad libitum access to chow and body composition was assessed using a magnetic resonance scanner (MRI) scanner (Bruker LF90II MRI scanner). The mice were then fed the Gubra Amylin Nonalcoholic steatohepatitis (GAN) diet, a 40 kcal% fat, 20 kcal% fructose, and 2% cholesterol diet (GAN, D09100310i; Research Diets) for 5 weeks, and blood glucose, plasma, and tissue samples were measured and collected as described for un‐anesthetized mice. In a separate experiment, female *Gcgr* ^−/−^ and *Gcgr* ^+/+^ mice, 7–16 weeks of age, were MRI scanned and then fed a high‐fat diet (HFD) diet, a 58 kcal% fat and sucrose, diet (D12331i; Research Diets) for 8 weeks. Following the 8 weeks the mice were again MRI scanned, blood glucose was measured, and plasma and tissue samples were collected as described for un‐anesthetized mice. Liver sections were stained with hematoxylin and eosin and steatosis was scored based on the following modified Kleiner (Kleiner et al., 2005) scoring system; steatosis (macro‐ and microvesicular) present in <5% of the hepatocytes = 0 point, 5%–33% = 1 point, 33%–66% = 2 point, and 66%–95% = 3 points.

### 2.3 Biochemical analysis

Triglyceride and glycerol concentrations were quantified using Serum Triglyceride Determination Kit (catalog no. TR0100‐1KT; Sigma‐Aldrich), except when measuring plasma triglyceride concentrations from the HFD fed mice, plasma and liver TG concentrations in the non‐fasted and fasted male *Gcgr* ^−/−^ and *Gcgr* ^+/+^ mice (shown in Figure S2), and when measuring plasma and liver triglyceride concentrations from the second group of HFD fed mice; here the Triglyceride Colorimetric Assay Kit (catalog no. 10010303; Cayman Chemical, MI, USA), the Triglyceride Quantification Colorimetric Kit (catalog no. MAK266‐1KT; Sigma‐Aldrich), and Triglyceride assay (catalog no. TR210, Randox) were used, respectively. Nonesterified fatty acid (NEFA) concentrations were measured with NEFA‐HR (R1 and R2) kit from Fujifilm Wako Chemicals and liver glycogen concentrations with EnzyChrom Glycogen Assay Kit (catalog no. E2GN‐100; BioAssay Systems, CA, USA). Plasma concentrations of glucagon were measured using a validated (Wewer Albrechtsen et al., 2016) low volume, two‐site enzyme immunoassay (catalog no. 10‐1281‐01; Mercodia, Upsala, Sweden). Fibroblast growth factor‐21 (FGF‐21) plasma concentrations were measured using mouse and rat FGF‐21 Elisa (catalog no. RD291108200R, BioVendor R&D, Czech Republic). Amino acid concentrations were measured using L‐Amino Acid Assay Kit (catalog no. ab65347; Abcam, USA). Total plasma Cholesterol concentrations were measured using a Total Cholesterol Assay (catalog no. CH200, Randox Laboratories Ltd), and the cholesterol content in plasma lipoproteins were measured as described in (Galsgaard et al., 2022).

### 2.4 Lipid extraction

Lipids from snap‐frozen liver tissue were extracted using a Triton™ X‐100 extraction buffer (3% Triton™ X‐100 (Catalog no. 10789704001; Sigma‐Aldrich) (25% solution in ethanol) in sodium acetate buffer (0.15 mol/L, pH 4.9)) as described in (Galsgaard et al., 2022).

### 2.5 Lipidomics

Lipids were extracted from liver samples (20 mg) from the mice feed the GAN diet and HFD using Folch extraction before transferring to HPLC vials as described previously (Hansen et al., 2024). A quality control (QC) sample was made by pooling 3 μL of each sample. Samples (0.5 μL) were injected using a Vanquish Horizon UPLC (Thermo Fisher Scientific) equipped with a Waters ACQUITY Premier CSH (2.1 × 100 mm, 1.7 μM) column operated at 55°C as described (Hansen et al., 2024). The flow from the UPLC was coupled to a TimsTOF Flex (Bruker) instrument for mass spectrometric analysis as described (Hansen et al., 2024). Compounds were annotated in Metaboscape (Bruker) using both an in‐built rule‐based annotation approach and using the LipidBlast MS2 library (Kind et al., 2013). Features were removed if their average signal were not >5× more abundant in the QC samples than blanks (water extraction). The signals were normalized to internal standards in the SPLASH mix before correction for signal drift using the statTarget R package (Luan et al., 2018), and signals were normalized using the QC samples. Peak intensities from each measured metabolite were scaled using Pareto scaling with MetabolAnalyze (version 1.3.1) and log2 transformed. Analysis of lipidomics data was done using the MetaboAnalyst 5.0 webtool (Pang et al., 2021). The lipodomics data will be made available.

### 2.6 Statistics

All statistical analyses were done in GraphPad Prism version 10.2.0 (La Jolla, California, USA). Groups were compared by unpaired *t*‐test, two‐way ANOVA, or mixed‐effects analysis as indicated in the figure legend. *p* < 0.05 was considered statistically significant.

## 3 RESULTS

### 3.1 Female glucagon receptor knockout mice show increased body weight

To determine the effects of permanent genetic deletion of the glucagon receptor on parameters of lipid metabolism in the non‐fasted and fasted state, we investigated non‐fasted and overnight fasted (16 h) female and male *Gcgr* ^−/−^ and *Gcgr* ^+/+^ mice.

Overnight fasting decreased blood glucose concentrations (*p* < 0.0001), body weight (*p* < 0.0001), and liver weights (*p* < 0.0001) in both female *Gcgr* ^−/−^ and *Gcgr* ^+/+^ mice.

**FIGURE 1:**
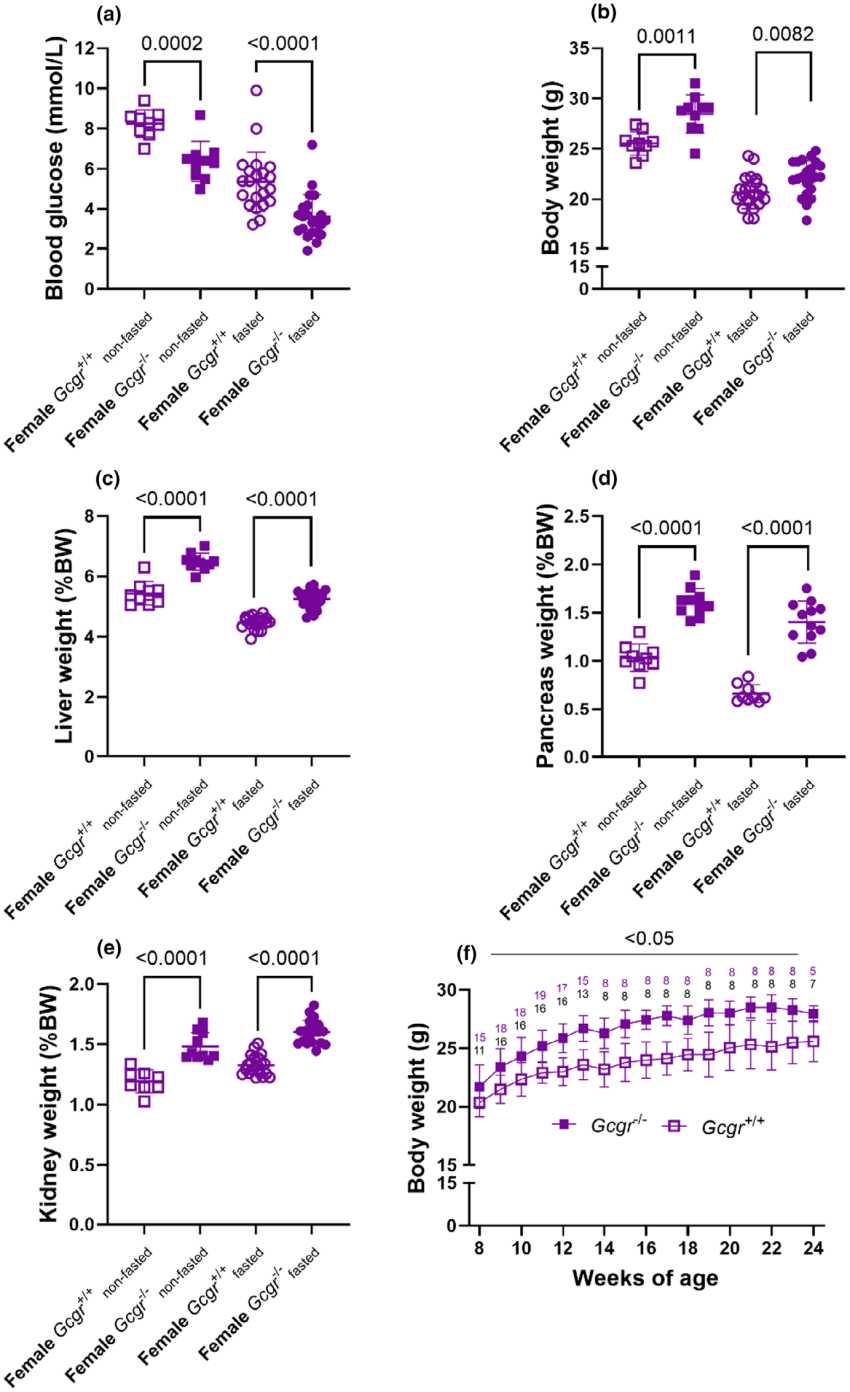
Glucagon receptor knockout female mice show increased body weight. (a) Blood glucose, (b) body weights, (c) liver weights, (d) pancreas weights, and (e) kidney weights in non‐fasted (squares) and overnight fasted (16 h) (circles) female wild‐type littermates (*Gcgr* ^+/+^, open symbols) and glucagon receptor knockout mice (*Gcgr* ^−/−^, closed symbols). Mice 14–18 weeks of age, *n* = 8–22. *p* value by unpaired *t*‐test. (f) Body weights of female *Gcgr* ^−/−^ and *Gcgr* ^+/+^ mice, the numbers in black indicate the number of *Gcgr* ^+/+^ and the numbers in magenta indicate the number of *Gcgr* ^−/−^ mice. *p* value by mixed‐effects analysis. Data shown as mean ± SD.

Blood glucose concentrations were lower in female *Gcgr* ^−/−^ compared to *Gcgr* ^+/+^ mice both in the non‐fasted and fasted state (*p* = 0.0002 and *p* < 0.0001, respectively) (Figure 1a). The body weight was increased in female *Gcgr* ^−/−^ compared to *Gcgr* ^+/+^ mice both in the non‐fasted and fasted state (*p* = 0.001 and *p* = 0.008, respectively) (Figure 1b). The glucagon receptor has been shown to be expressed in three organs; the liver, pancreas, and kidney (Bomholt et al., 2021). We therefore assessed the weight of these organs and found the weight of all three organs to be increased in female *Gcgr* ^−/−^ mice compared to *Gcgr* ^+/+^ mice both in the non‐fasted and fasted state (*p* < 0.0001) (Figure 1c–e). The increased pancreas weight is most likely due hyperaminoacidemia mediated alpha‐cell hyperplasia (Dean et al., 2017; Gelling et al., 2003). Similar results were obtained in male mice (Figure S1).

To further investigate the difference observed in body weight, we weighed a separate cohort of female *Gcgr* ^−/−^ and *Gcgr* ^+/+^ mice once weekly and found the body weights to be increased at all ages ranging from 7 to 24 weeks (Figure 1f).

**FIGURE 2:**
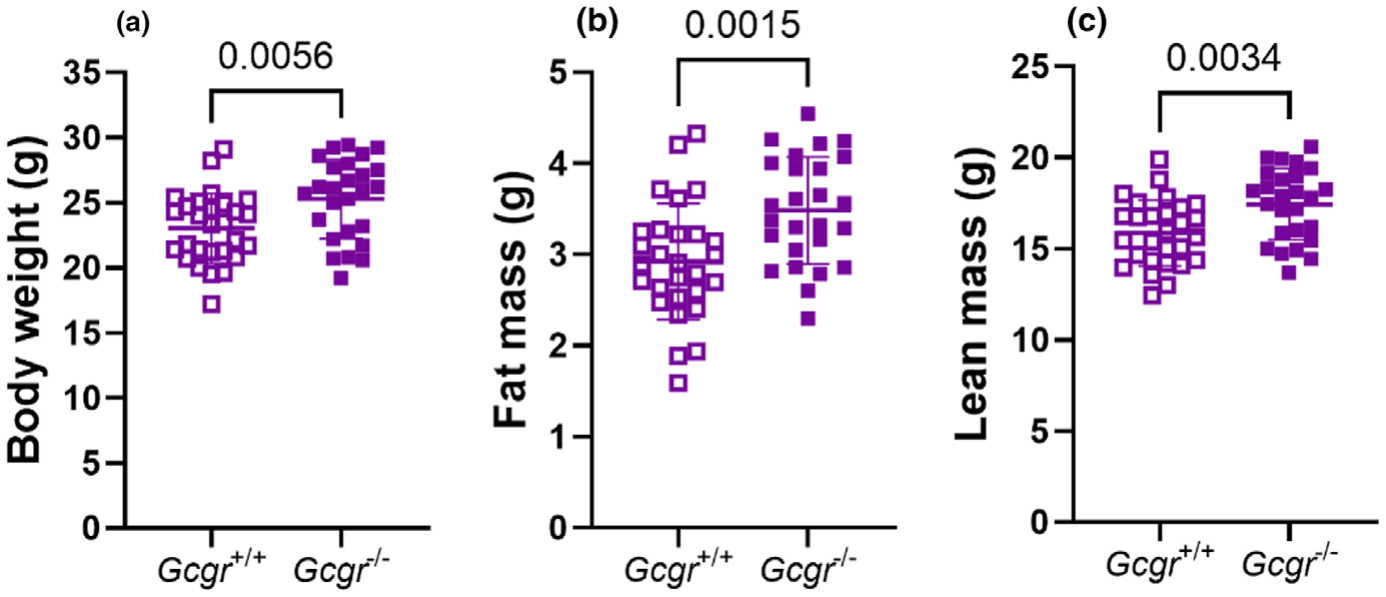
Female glucagon receptor knockout mice show an increased absolute lean and fat mass. (a) Body weights, (b) absolute fat mass, and (c) absolute lean mass in female wild‐type littermates (*Gcgr* ^+/+^, open symbols) and glucagon receptor knockout mice (*Gcgr* ^−/−^, closed symbols). Data shown as mean ± SD, *n* = 26–28, mice 7–18 weeks of age. *p* value by unpaired *t*‐test.

To investigate the details behind the increased body weight of *Gcgr* ^−/−^ mice, we assessed the body composition using MR scans in a group of female mice in the non‐fasted state (following the scan these mice were fed either a GAN diet or HFD). The *Gcgr* ^−/−^ mice showed increased body weight (*p* = 0.006) (Figure 2a), and both the absolute fat mass (Figure 2b) and lean mass (Figure 2c) were higher in *Gcgr* ^−/−^ mice compared to *Gcgr* ^+/+^ mice (*p* = 0.002 and *p* = 0.003, respectively).

### 3.2 Fasting‐induced steatosis masks the increase in liver triglycerides observed in non‐fasted female glucagon receptor knockout mice

#### 3.2.1 Plasma parameters

Overnight fasting increased plasma nonesterified fatty acid (NEFA) (*p* < 0.0005) and liver TG concentrations (*p* < 0.0003) while decreasing liver glycogen concentrations (*p* < 0.0001) in both female *Gcgr* ^−/−^ and *Gcgr* ^+/+^ mice.

**FIGURE 3:**
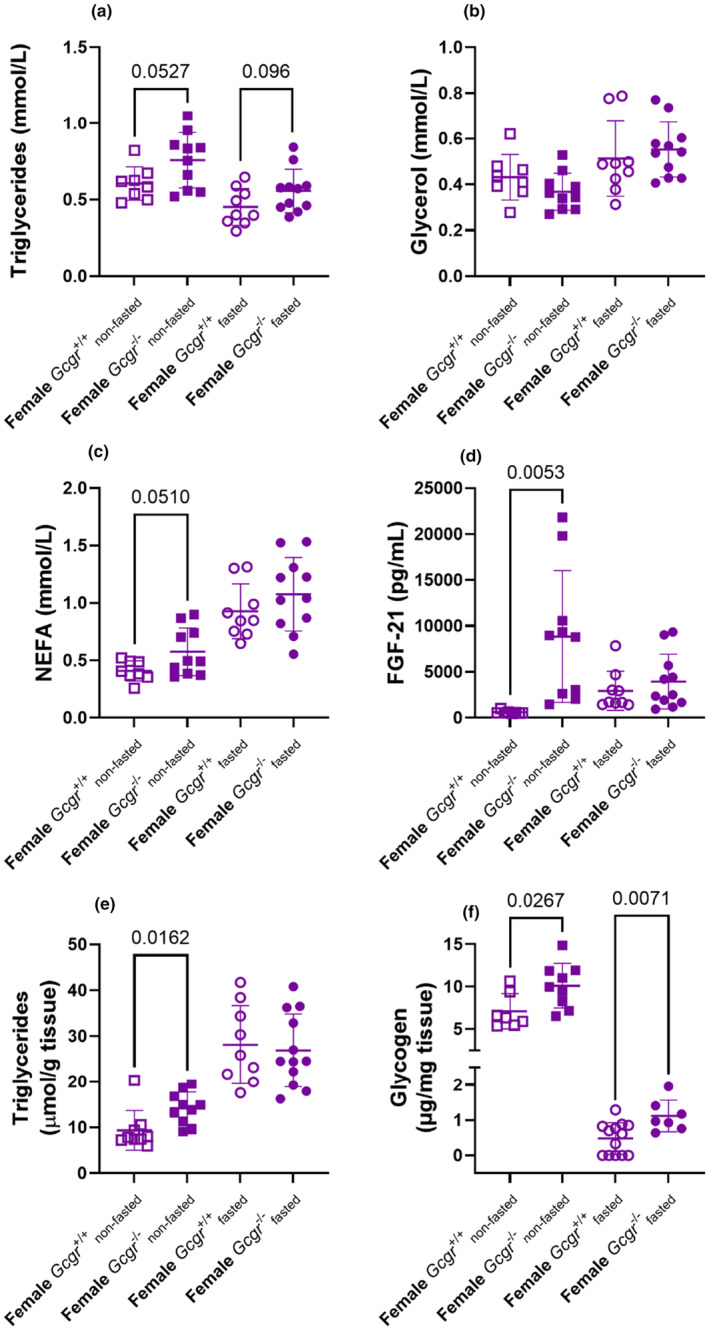
Non‐fasted female glucagon receptor knockout mice show increased liver triglyceride concentrations. (a) Plasma triglyceride, (b) glycerol, (c) nonesterified fatty acid (NEFA), (d) fibroblast growth factor 21 (FGF‐21), (e) liver triglyceride, and (f) liver glycogen (five of the measurements were under the detection limit and shown as 0 μg/mg) concentrations in non‐fasted (squares) and overnight fasted (16 h) (circles) female wild‐type littermates (*Gcgr* ^+/+^, open symbols) and glucagon receptor knockout mice (*Gcgr* ^−/−^, closed symbols). Data shown as mean ± SD, *n* = 7–12, mice 14–18 weeks of age. *p* value by unpaired *t*‐test.

In both the non‐fasted and fasted state, plasma TG concentrations were numerically higher in *Gcgr* ^−/−^ than in *Gcgr* ^+/+^ mice (*p* = 0.053 and *p* = 0.096, respectively) (Figure 3a). Plasma glycerol concentrations were similar in female *Gcgr* ^−/−^ and *Gcgr* ^+/+^ mice both in the non‐fasted and fasted state (*p* > 0.1) (Figure 3b). When compared to female *Gcgr* ^+/+^ mice, plasma NEFA concentrations were numerically higher in female *Gcgr* ^−/−^ mice in the non‐fasted state (*p* = 0.051), but not in the fasted state (*p* = 0.2) (Figure 3c). Fibroblast growth factor 21 (FGF‐21) regulates lipid metabolism by increasing lipolysis in adipocytes and in the liver (Inagaki et al., 2007). We therefore assessed plasma concentrations of FGF‐21 and found that in the non‐fasted state plasma FGF‐21 concentrations were increased in *Gcgr* ^−/−^ mice (*p* = 0.005), but in the fasted state plasma FGF‐21 concentrations were similar to those of *Gcgr* ^+/+^ mice (*p* = 0.4) (Figure 3d). In female *Gcgr* ^+/+^ mice, fasting increased FGF‐21 concentrations (*p* = 0.008), whereas in *Gcgr* ^−/−^ mice fasting, if anything, lowered FGF‐21 concentrations (*p* = 0.051).

#### 3.2.2 Liver parameters

In the non‐fasted state, liver TG concentrations were increased in female *Gcgr* ^−/−^ mice (*p* = 0.02), whereas in the fasted state liver TG concentrations were similar in *Gcgr* ^−/−^ and *Gcgr* ^+/+^ mice (*p* = 0.7) but increased compared to the non‐fasted state (Figure 3e), suggesting that fasting induced‐steatosis masks the steatosis‐promoting effect of permanent genetic deletion of the glucagon receptor. Liver glycogen concentrations were increased in *Gcgr* ^−/−^ mice compared to female *Gcgr* ^+/+^ mice in the non‐fasted (*p* = 0.03) and fasted state (*p* = 0.007) (Figure 3f).

In male mice similar results were obtained with the exception of plasma TG concentrations being increased in non‐fasted male *Gcgr* ^+/+^ compared to *Gcgr* ^−/−^ mice (*p* = 0.03), and the increased in liver TG concentrations being numerically higher in male *Gcgr* ^−/−^ compared to *Gcgr* ^+/+^ mice (*p* = 0.0579) (Figure S2).

### 3.3 Glucagon receptor knockout mice show hypercholesterolemia

**FIGURE 4:**
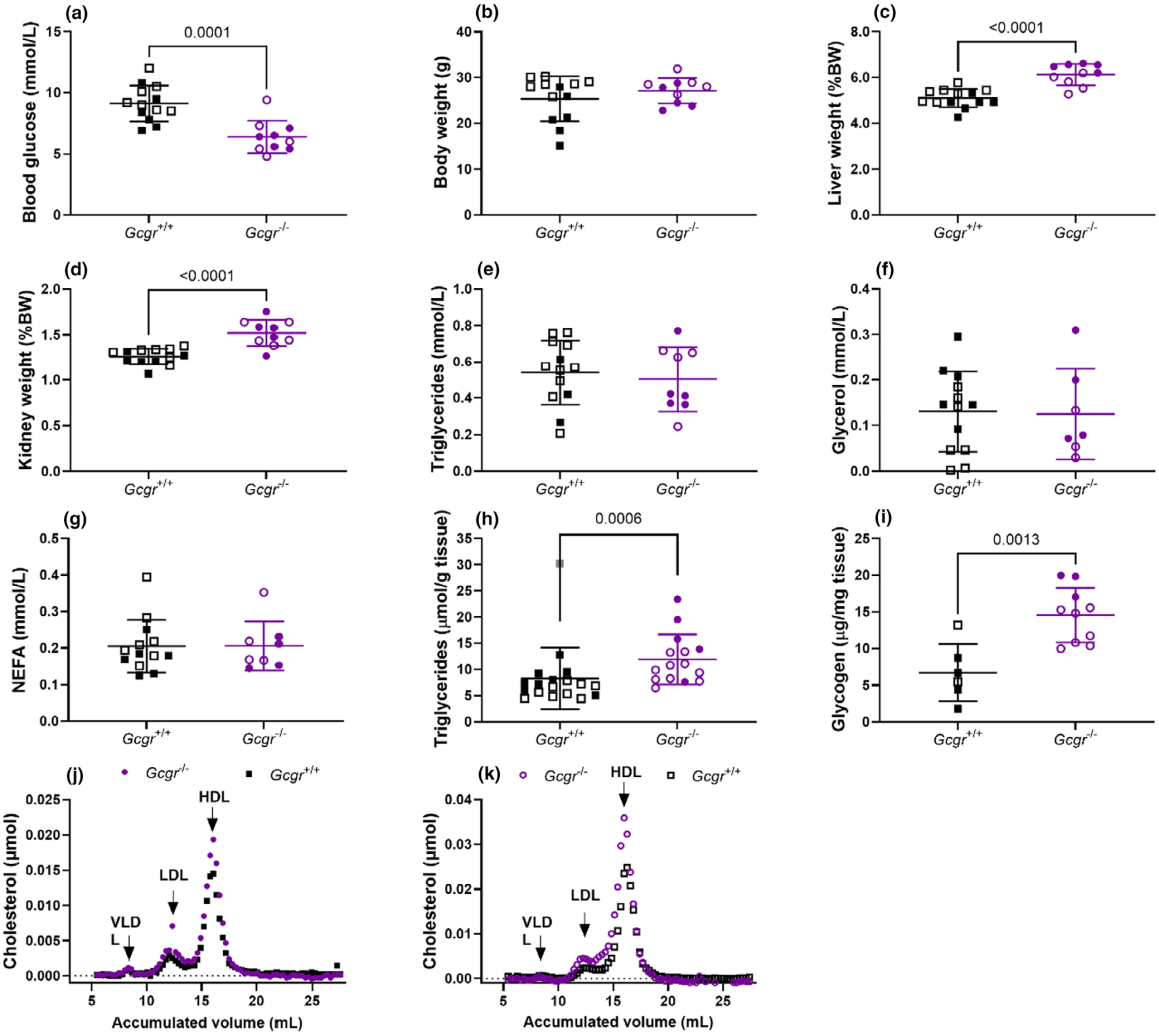
Non‐fasted glucagon receptor knockout mice show hypercholesterolemia. (a) Blood glucose, (b) body weights, (c) liver weights, (d) kidney weights, (e) plasma triglyceride, (f) glycerol, (g) nonesterified fatty acid (NEFA), (h) liver triglyceride (one mouse, shown in gray, had an abnormally high liver triglyceride concentration (>30 μmol/g tissue) and was excluded), and (i) liver glycogen concentrations in wild‐type littermates (*Gcgr* ^+/+^, black squares) and glucagon receptor knockout mice (*Gcgr* ^−/−^, purple circles). Values obtained from female mice are shown in closed symbols and values obtained from male mice are shown in open symbols. Plasma cholesterol profiles from female (j) and male (k) *Gcgr* ^−/−^ and *Gcgr* ^+/+^ mice. Very‐low density lipoprotein (VLDL), low density lipoprotein (LDL), and high density lipoprotein (HDL). Data shown as mean ± SD, *n* = 4–15, mice 7–16 weeks of age. *p* value by unpaired *t*‐test.

To further investigate the lipid metabolism in *Gcgr* ^−/−^ mice, we investigated another cohort of non‐fasted anesthetized *Gcgr* ^−/−^ and *Gcgr* ^+/+^ female and male mice. Blood glucose concentrations in *Gcgr* ^−/−^ mice were, as expected, decreased compared to *Gcgr* ^+/+^ mice (*p* = 0.0001) (Figure 4a). Body weights were similar in *Gcgr* ^−/−^ and *Gcgr* ^+/+^ mice (females; *p* = 0.1, males; *p* = 0.9) (Figure 4b), possibly because of the small number of mice investigated. Liver and kidney weights were again increased in *Gcgr* ^−/−^ compared to *Gcgr* ^+/+^ mice (both *p* < 0.0001) (Figure 4c,d). No difference in plasma TG, glycerol, or NEFA concentrations were found between the genotypes (*p* > 0.6, *p* > 0.9, and *p* > 0.9, respectively) (Figure 4e–g). Liver TG and glycogen concentrations were increased in *Gcgr* ^−/−^ compared to *Gcgr* ^+/+^ mice (*p* = 0.0006 and *p* = 0.001, respectively) (Figure 4h,i). When liver TG concentrations were stratified by sex, both female and male *Gcgr* ^−/−^ mice had increased liver TG compared to *Gcgr* ^+/+^ mice of the same sex (females; *p* = 0.006, males; *p* = 0.0003). Finally, we found plasma concentrations of low‐density lipoprotein (LDL) and high‐density lipoprotein (HDL) to be increased in *Gcgr* ^−/−^ when compared to *Gcgr* ^+/+^ mice (Figure 4j,k).

### 3.4 Glucagon receptor knockout mice show lipid intolerance

**FIGURE 5:**
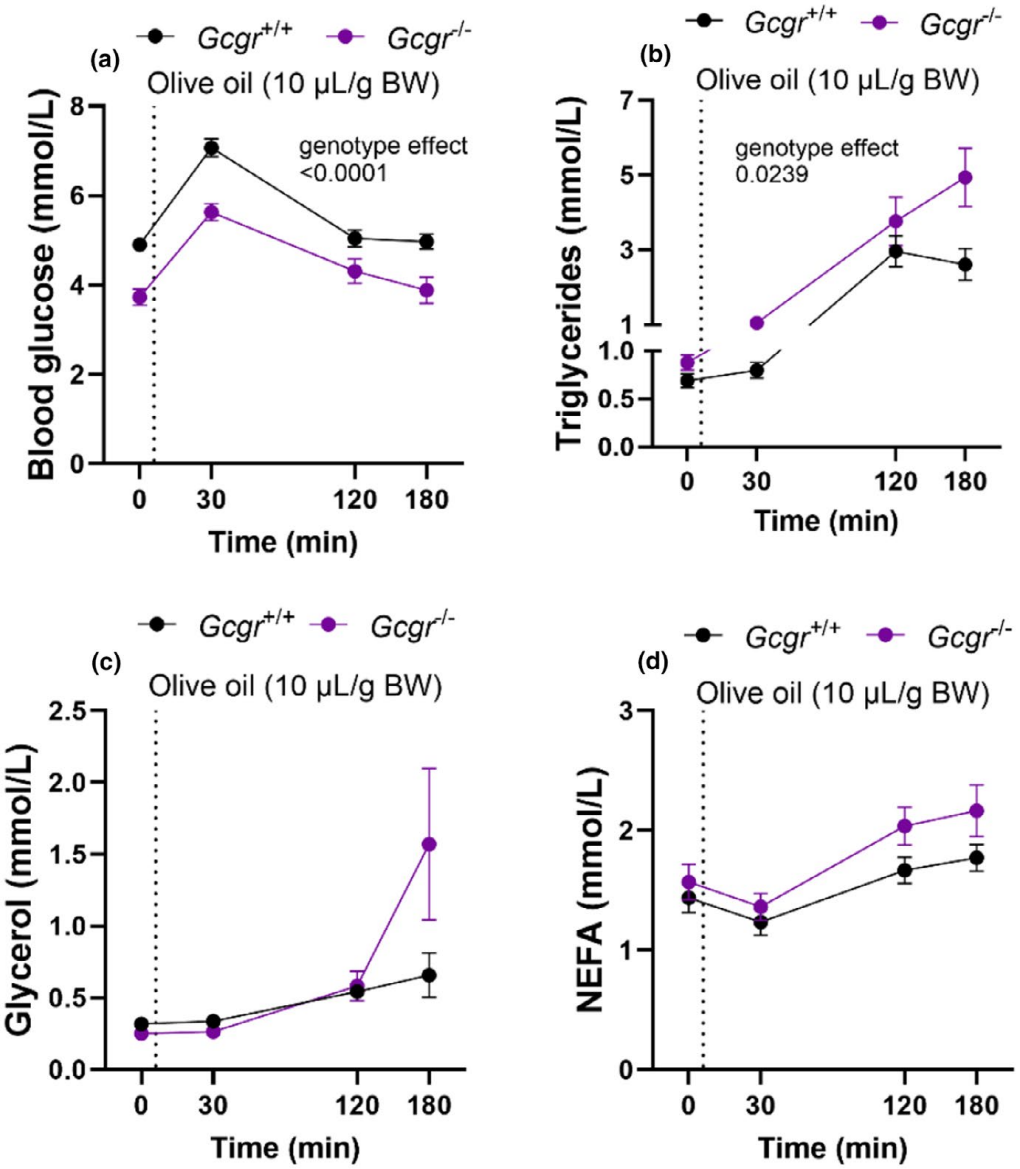
Glucagon receptor knockout mice show lipid intolerance. (a) Blood glucose, (b) plasma triglyceride (c) glycerol, (d) non‐esterified fatty acid (NEFA) concentrations in glucagon receptor knockout mice (*Gcgr* ^−/−^, purplee circles) and their wild‐type littermates (*Gcgr* ^+/+^, black circles) during a lipid tolerance test (olive oil, 10 μL/g body weight). Data shown as mean ± SEM, *n* = 9–16, mice 10–14 weeks of age. *p* value by (a) two‐way ANOVA or (b–d) mixed‐effects analysis.

To investigate postprandial lipid metabolism, we challenged a mixed cohort of male and female *Gcgr* ^−/−^ and *Gcgr* ^+/+^ mice with an oral lipid tolerance test (olive oil, 10 μL/g body weight via oral gavage; OLTT). The mice were overnight fasted, and fasting blood glucose concentrations (0 min time‐point of the OLTT) were decreased in *Gcgr* ^−/−^ mice compared to *Gcgr* ^+/+^ mice (*p* < 0.0001), while fasting TG and glycerol concentrations were numerically increased in *Gcgr* ^−/−^ mice (*p* = 0.09 and *p* = 0.09), whereas plasma NEFA concentrations were similar in *Gcgr* ^−/−^ and *Gcgr* ^+/+^ mice (*p* = 0.5) (Figure 5).

During the OLTT the *Gcgr* ^−/−^ mice had lower blood glucose concentrations than *Gcgr* ^+/+^ mice (genotype effect *p* < 0.0001) (Figure 5a). After lipid administration plasma TG concentrations were increased in *Gcgr* ^−/−^ compared to mice *Gcgr* ^+/+^ (genotype effect *p* > 0.03) (Figure 5b). Glycerol (genotype effect *p* > 0.2) (Figure 5c) and NEFA (genotype effect *p* > 0.09) (Figure 5d) concentrations were similar during the OLTT. Female and male mice are shown individually Figure S3.

### 3.5 Female glucagon receptor knockout mice show dyslipidemia and increased liver fat when challenged with GAN diet and HFD

To investigate whether the disturbances observed in lipid metabolism in *Gcgr* ^−/−^ mice would be augmented when challenged with a diet high in cholesterol and fat, and to investigated if mice lacking glucagon receptor signaling were able to tolerate such a diet, we challenged female *Gcgr* ^−/−^ and *Gcgr* ^+/+^ mice with a GAN diet (the Gubra Amylin Nonalcoholic steatohepatitis (GAN) diet consisting of 40 kcal% fat, 20 kcal% fructose, and 2% cholesterol) for 5 weeks and in separate experiments female *Gcgr* ^−/−^ and *Gcgr* ^+/+^ mice were challenged with a HFD (58 kcal% fat and sucrose) for 8 weeks.

**FIGURE 6:**
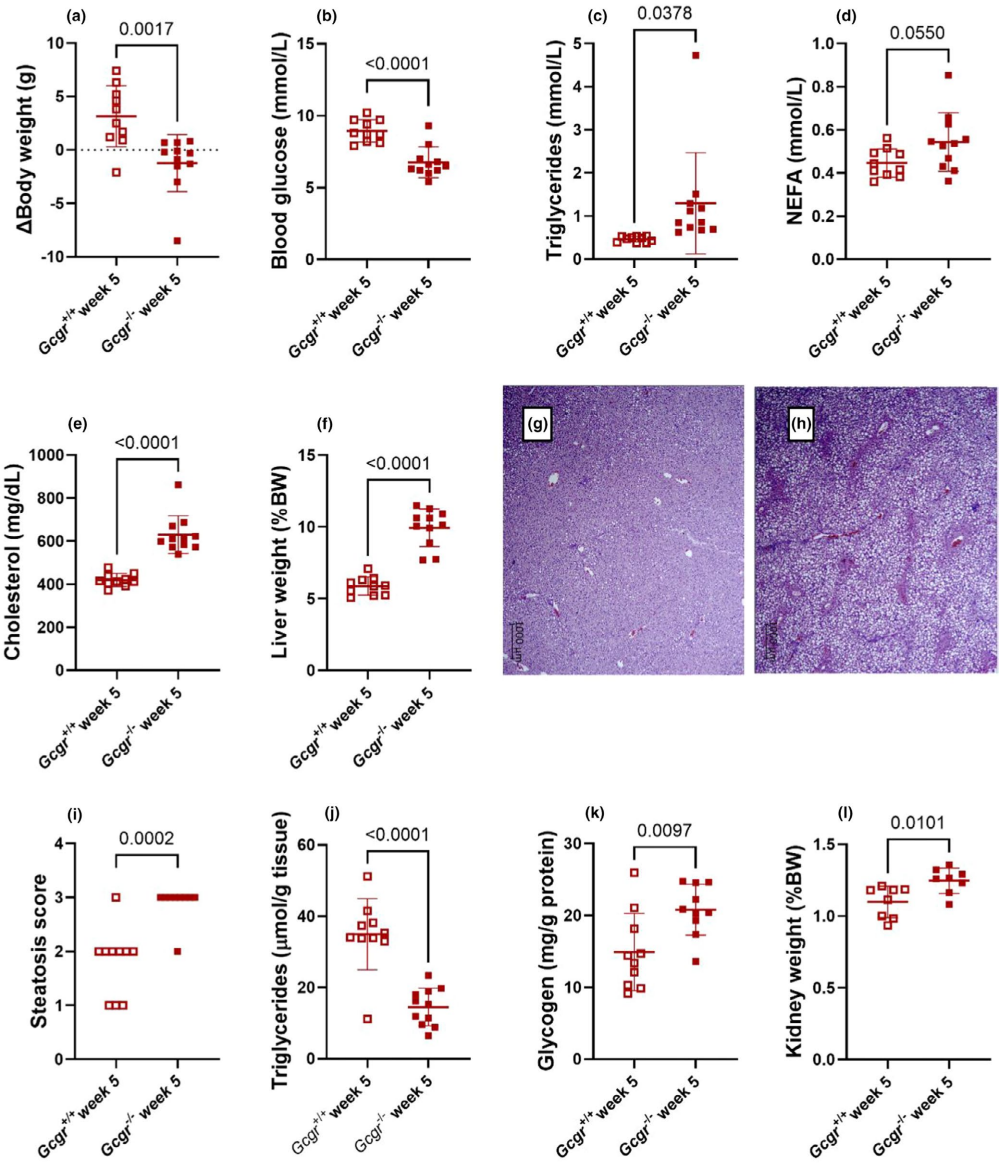
Female glucagon receptor knockout mice show dyslipidemia and increased liver fat when challenged with a GAN diet. (a) ΔBody weights, (b) blood glucose, (c) plasma triglyceride, (d) non‐esterified free fatty acid (NEFA), (e) cholesterol, (f) liver weights, (g) representive H&E staining of wild‐type littermates (*Gcgr* ^+/+^) and (h) glucagon receptor knockout mice (*Gcgr* ^−/−^) livers, (i) steatosis score, (j) liver triglyceride concentrations, (k) liver glycogen, and (l) kidney weights in female *Gcgr* ^+/+^ (open symbols) and *Gcgr* ^−/−^ (closed symbols) mice after 5 weeks of Gubra Amylin Nonalcoholic steatohepatitis diet feeding. Data shown as mean ± SD, *n* = 10–12, mice 22–29 weeks of age. *p* value by unpaired *t*‐test.

Before the 5 weeks of GAN diet feeding, the female *Gcgr* ^−/−^ mice had a higher body weight than *Gcgr* ^+/+^ mice (*p* = 0.03; these data are included in Figure 2a), however after 5 weeks of GAN diet feeding the *Gcgr* ^−/−^ mice had lost weight while the *Gcgr* ^+/+^ mice had gained weight (−1.2 ± 0.8 vs. 3.2 ± 0.9 g) (Figure 6a). Blood glucose concentrations were lower in the *Gcgr* ^−/−^ mice before (data not shown) and after the diet (Figure 6b) (both *p* < 0.0001). After the 5 weeks of diet, plasma TG concentrations were increased in the *Gcgr* ^−/−^ compared to *Gcgr* ^+/+^ mice (*p* = 0.04) (Figure 6c) (also after excluding the highest value measured in the *Gcgr* ^−/−^ mice (*p* < 0.0001)). Plasma NEFA (*p* = 0.055) (Figure 6d) and total cholesterol (*p* < 0.0001) (Figure 6e) concentrations were also increased in the *Gcgr* ^−/−^ compared to *Gcgr* ^+/+^ mice. After the diet, the liver weights of the *Gcgr* ^−/−^ mice were increased compared to *Gcgr* ^+/+^ mice (*p* < 0.0001) (Figure 6f). H&E staining's of the livers revealed that *Gcgr* ^−/−^ mice had a higher degree of hepatic lipid accumulation compared to *Gcgr* ^+/+^ mice (representative sections shown in Figure 6g,h and steatosis score in Figure 6i). When quantifying the liver TG content enzymatically, the histology results were not confirmed as *Gcgr* ^−/−^ mice showed decreased amounts of extractable liver TGs compared to *Gcgr* ^+/+^ mice (*p* < 0.0001) (Figure 6j). Consistent with our previous data, glycogen concentrations were increased after the GAN diet in the livers of *Gcgr* ^−/−^ mice (*p* = 0.001) (Figure 6k). Kidney weights were again increased in *Gcgr* ^−/−^ mice compared to *Gcgr* ^+/+^ mice after the GAN diet (*p* = 0.01) (Figure 6l).

**FIGURE 7:**
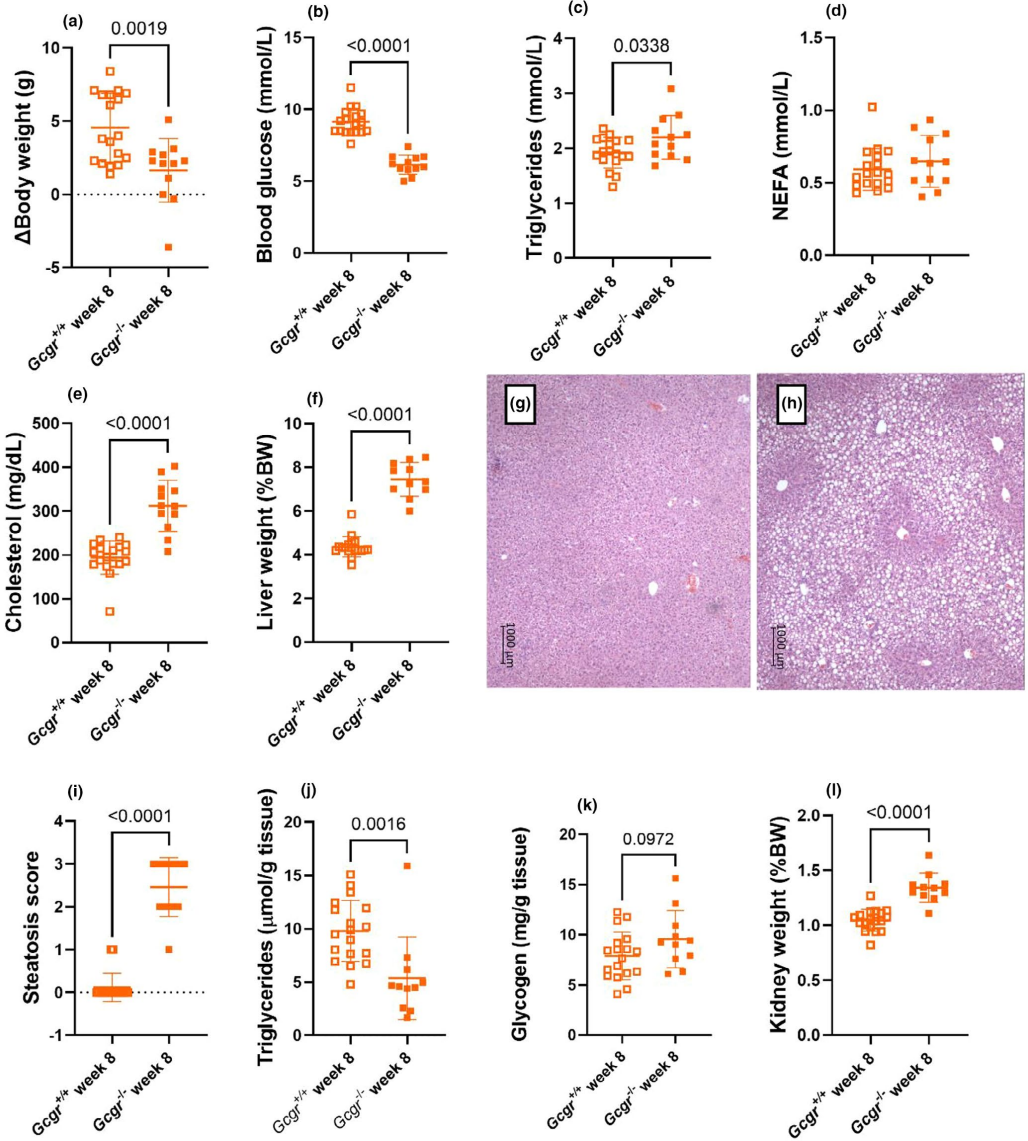
Female glucagon receptor knockout mice show dyslipidemia and increased liver fat when challenged with a high‐fat diet. (a) ΔBody weights, (b) blood glucose, (c) plasma triglyceride, (d) nonesterified free fatty acid (NEFA), (e) cholesterol, (f) liver weights, (g) representive H&E staining of wild‐type littermates (*Gcgr* ^+/+^) and (h) glucagon receptor knockout mice (*Gcgr* ^−/−^) livers, (i) steatosis score, (j) liver triglyceride concentrations, (k) liver glycogen concentrations, and (l) kidney weights in female *Gcgr* ^+/+^ (open symbols) and *Gcgr* ^−/−^ (closed symbols) mice 8 weeks after high‐fat diet feeding. Data shown as mean ± SD, *n* = 12–18, mice 16–25 weeks of age. *p* value by unpaired *t*‐test.

The results observed after the GAN diet were replicated in female *Gcgr* ^−/−^ and *Gcgr* ^+/+^ mice challenged with a HFD for 8 weeks (Figure 7). Again, when quantifying the liver TG content enzymatically, the histology results were not confirmed as *Gcgr* ^−/−^ mice showed decreased amounts of extractable liver TGs compared to *Gcgr* ^+/+^ mice (*p* < 0.002) (Figure 7j). During the 8 weeks of HFD the *Gcgr* ^−/−^ gained significantly less fat mass compared to *Gcgr* ^+/+^ (*p* = 0.0005) and tended to also gain less lean mass (*p* = 0.0517) (Figure S4).

We repeated the HFD study, and fed a small cohort of female and male *Gcgr* ^−/−^ and *Gcgr* ^+/+^ mice the same HFD for 8 weeks. The results were replicated except the change in body weight, plasma TG, plasma NEFA concentrations, liver TG, and steatosis score not being significantly different between *Gcgr* ^−/−^ and *Gcgr* ^+/+^ mice. Additionally, we noticed that male *Gcgr* ^−/−^ mice showed hypercholesterolemia, increased liver weight, and steatosis to a lesser degree than female *Gcgr* ^−/−^ mice; however, our study was not powered to detect significant sex differences (Figure S5).

### 3.6 Lipidomics analysis reveals significant difference in the lipidome of GAN diet and HFD fed Gcgr^−/−^ and Gcgr^+/+^ female mice

To investigate the discrepancy between the histological manifestation of hepatic lipid droplets and the enzymatic quantification of liver TG following the GAN diet and HFD in detail we conducted a mass‐spectromety based lipidomics analysis of liver biopsies from the *Gcgr* ^*−/−*^ and *Gcgr* ^*+/+*^ female mice fed a GAN diet and HFD. When comparing all four groups (*Gcgr* ^*−/−*^ GAN diet, *Gcgr* ^*+/+*^ GAN diet, *Gcgr* ^*−/−*^ HFD, and *Gcgr* ^*+/+*^ HFD) the diets completely separated the four groups into two, thus revealing that the mice fed the GAN diet have a different lipidome that the mice fed the HFD (Figure S6a). Not surprisingly sterols were significantly upregulated in the livers of mice receiving the GAN diet (Figure S6b). Only six lipid species showed a significant effect due to genotype alone and four lipid species showed a significant interaction effect between genotype and diet (Figure S6c).

**FIGURE 8:**
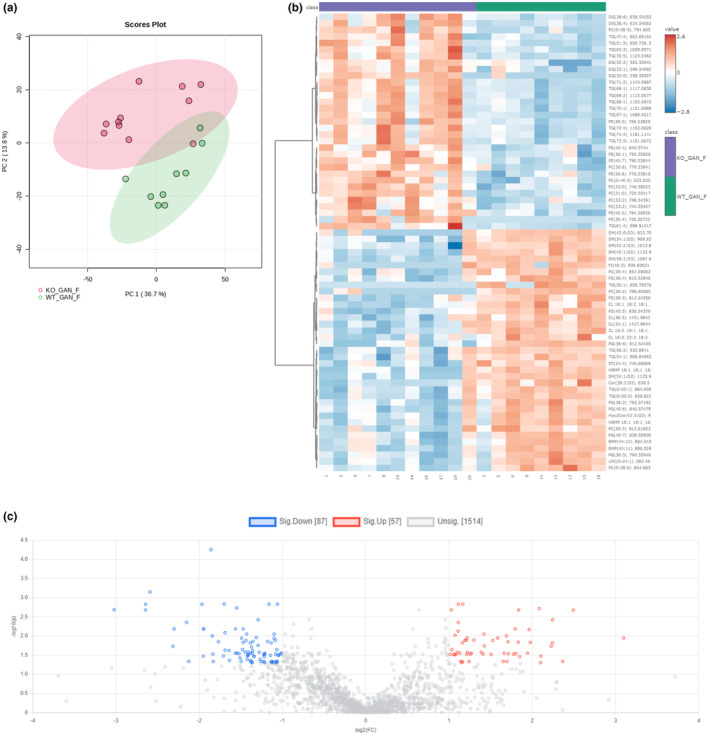
Lipidomics analysis reveals significant difference in the lipidome of female glucagon receptor knockout mice challenged with a GAN diet. (a) Principal component analysis plot, (b) heat map, and (c) volcano plot (fold change treshold 2.0, *p* value treshold 0.05 false discovery rate corrected, direction of comparison KO_GAN_F/ WT_GAN_F) showing differencences in the lipidome of female glucagon receptor knockout mice (KO_GAN_F) and wild‐type littermates (WT_GAN_F) after 5 weeks of Gubra Amylin Nonalcoholic (GAN) steatohepatitis diet feeding.

When comparing the *Gcgr* ^*−/−*^ and *Gcgr* ^*+/+*^ fed the GAN diet, the mice clustered together based on genotype (Figure 8a). The *Gcgr* ^*−/−*^ showed enrichment of specific diacylglycerols, TG, phosphatidylcholine, phosphatidylethanolamine, and phosphatidylserine species whereas sphingomyelin and cardiolipin species among others were less abundant (Figure 8b). In the *Gcgr* ^*−/−*^ 87 and 57 lipid species were found to be significantly down‐ and up‐regulated, respectively (Figure 8c), the 20 most down‐ and up‐regulated species are shown in Table S1.

**FIGURE 9:**
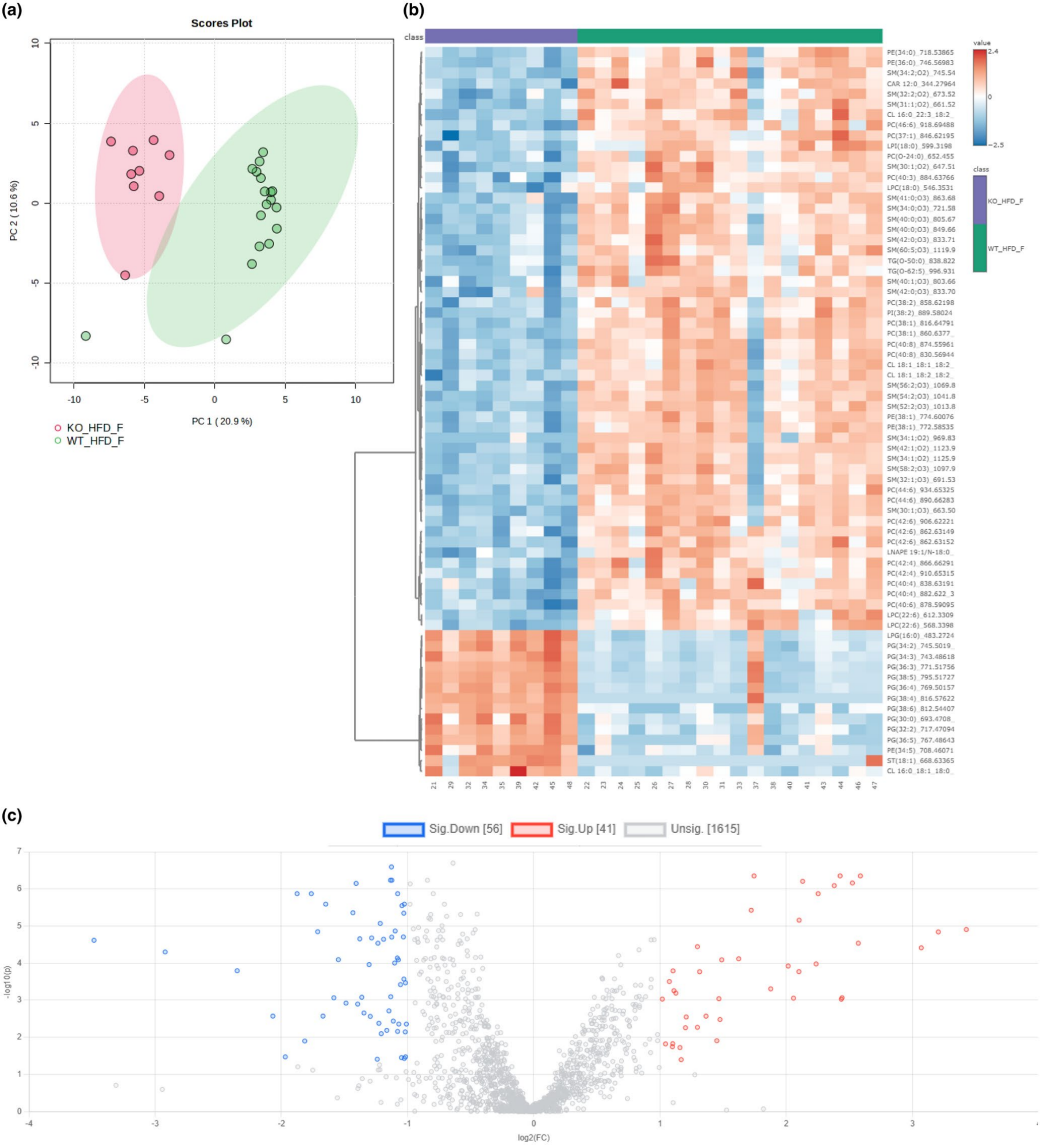
Lipidomics analysis reveals significant difference in the lipidome of female glucagon receptor knockout mice challenged with high‐fat diet. (a) Principal component analysis plot, (b) heat map, and (c) volcano plot (fold change treshold 2.0, *p* value treshold 0.05 false discovery rate corrected, direction of comparison KO_HFD_F/ WT_HFD_F) showing differencences in the lipodome of female glucagon receptor knockout mice (KO_HFD_F) and wild‐type littermates (WT_HFD_F) after 8 weeks of high‐fat diet feeding.

When comparing the *Gcgr* ^*−/−*^ and *Gcgr* ^*+/+*^ fed the HFD mice, the mice again clustered together based on genotype (Figure 9a). The *Gcgr* ^*−/−*^ mice showed enrichment of specific TG and sphingomyelin species among others whereas the *Gcgr* ^*+/+*^ showed an enrichment of diacylglycerol species among others (Figure 9b). In the *Gcgr* ^*−/−*^ 56 and 41 lipid species were found to be significantly down‐ and up‐regulated, respectively (Figure 9c), the 20 most down‐ and up‐regulated species are shown in Table S2.

### 3.7 Female glucagon receptor knockout mice show a decreased urea index

**FIGURE 10:**
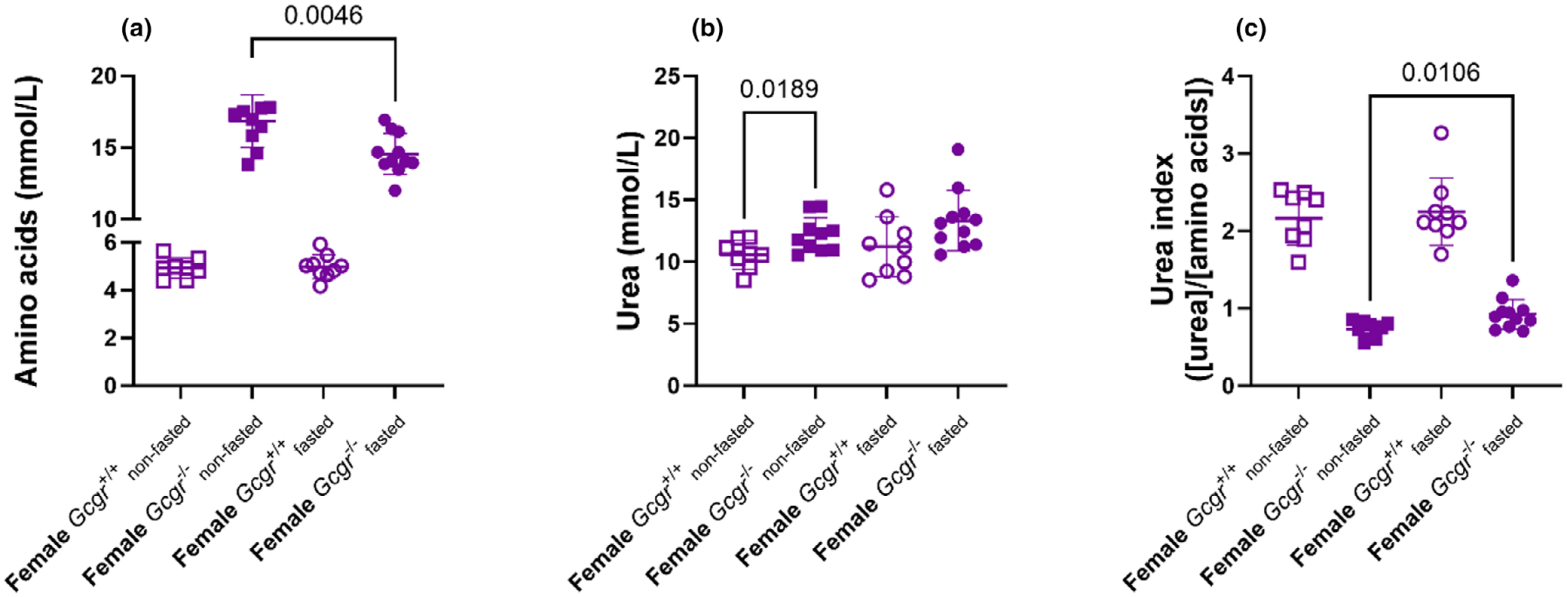
Female glucagon receptor knockout mice show a decreased urea index. (a) Plasma amino acid, (b) urea concentrations, and (c) urea index in non‐fasted (squares) and overnight fasted (16 h) (circles) female wild‐type littermates (*Gcgr* ^+/+^, open symbols) and glucagon receptor knockout mice (*Gcgr* ^−/−^, closed symbols). Data shown as mean ± SD, *n* = 8–12, mice 14–18 weeks of age. *p* value by unpaired *t*‐test.

To investigate how *Gcgr* ^−/−^ mice adapt protein metabolism in response to fasting we assessed plasma amino acid and urea concentrations in non‐fasted and fasted *Gcgr* ^−/−^ and *Gcgr* ^+/+^ mice. Plasma amino acid concentrations were increased in non‐fasted (*p* < 0.0001) and fasted (*p* < 0.0001) female *Gcgr* ^−/−^ compared to *Gcgr* ^+/+^ mice (Figure 10a). In female *Gcgr* ^+/+^ mice, plasma amino acid concentrations did not differ between the non‐fasted and fasted state (*p* = 0.8), whereas in *Gcgr* ^−/−^ mice plasma amino acid concentrations decreased upon fasting (*p* = 0.005). Plasma urea concentrations were increased in non‐fasted (*p* = 0.02) and fasted (0.07) *Gcgr* ^−/−^ female mice compared to *Gcgr* ^+/+^ mice (Figure 10b). In neither genotype did plasma urea concentrations change upon fasting (*p* > 0.2). The plasma urea index ([urea]/[amino acids]) was decreased in non‐fasted (*p* < 0.0001) and fasted (*p* < 0.0001) *Gcgr* ^−/−^ female mice compared to *Gcgr* ^+/+^ mice (Figure 10c). In *Gcgr* ^+/+^ mice, the urea index did not differ between the non‐fasted and fasted state (*p* = 0.7), whereas in *Gcgr* ^−/−^ mice the urea index increased upon fasting (*p* = 0.01).

Similar results were obtained in male mice (Figure S7), with the exception of plasma urea concentrations being similar in non‐fasted (*p* = 0.2) and fasted (0.9) *Gcgr* ^−/−^ and *Gcgr* ^+/+^ male mice, and that in *Gcgr* ^−/−^ mice plasma urea concentrations increased upon fasting (*p* = 0.04).

## 4 DISCUSSION

We here show that female *Gcgr* ^−/−^ mice challenged with a GAN diet (promoting MASH) and a HFD (promoting hepatic steatosis) were resistant to weight gain but prone to steatosis and dyslipidemia. When fed a chow diet, non‐fasted *Gcgr* ^−/−^ mice showed hypercholesterolemia (increased LDL and HDL plasma concentrations) and increased liver fat. The latter was masked by fasting‐induced steatosis. The increase in liver TG concentrations was borderline significant (*p* = 0.0579) in non‐fasted male *Gcgr* ^−/−^ mice (Figure S2), but significant (*p* < 0.02) in the remaining groups. Additionally, fasted *Gcgr* ^−/−^ mice, fed a standard chow diet, show impaired lipid tolerance. In response to overnight (16 h) fasting, *Gcgr* ^−/−^ mice, fed a standard chow diet, were able to decrease hepatic glycogen content and did not become hypoglycemic. Upon fasting, plasma amino acid concentrations decreased in *Gcgr* ^−/−^ mice while the urea‐index increased (suggesting increased substrate induced ureagenesis and thus increased amino acid catabolism), whereas these parameters remained unchanged in *Gcgr* ^+/+^ mice. Taken together, our data suggest that in *Gcgr* ^−/−^ mice lipid metabolism is impaired and the impairment becomes augmented when the mice are challenge with a GAN diet and HFD, thus supporting glucagon as an important physiological regulator of not just glucose and amino acid, but also lipid metabolism.

During fasting, lipolysis in adipose tissue is increased, resulting in increased flux of lipids to the liver causing fasting‐induced steatosis. The TGs consequently stored in the liver are used for energy, and glucagon may be required for this adaptive response to fasting in mice (Longuet et al., 2008). A study showed that *Gcgr* ^−/−^ mice (both sexes) fasted for 16 h failed to increase hepatic lipid oxidation and showed an increase in hepatic lipid secretion, resulting in increased plasma TG concentrations (Longuet et al., 2008). In contrast, fasted and non‐fasted TG plasma concentrations were found to be similar in *Gcgr* ^−/−^ and *Gcgr* ^+/+^ male mice, while fed TG plasma concentrations were 60% decreased in female *Gcgr* ^−/−^ mice (Gelling et al., 2003). Similar plasma TG and slightly elevated plasma cholesterol concentrations were found in male and female *Gcgr* ^−/−^ mice following a 14–16 h fast (Parker et al., 2002). This is consistent with the clinical finding that treatment with glucagon receptor antagonists increased plasma LDL, HDL, and TG concentrations in individuals with type 2 diabetes (Kazda et al., 2017). We also found non‐fasted *Gcgr* ^−/−^ mice, both males and females, to have increased LDL and HDL plasma concentrations. Similarly, plasma LDL was increased in non‐fasted *Gcgr* ^−/−^ mice (Gelling et al., 2003). In addition, *Gcgr* ^−/−^ mice showed impaired lipid tolerance during an OLTT following an overnight fast, as also shown previously (Galsgaard et al., 2022). We found a tendency to plasma TG concentrations to be numerically higher in *Gcgr* ^−/−^ mice, however this was dependent on the sex and fasting state of the mice. Male *Gcgr* ^−/−^ mice fed a HFD have been shown to be prone to steatosis (Longuet et al., 2008), and show a greater hepatic lipid accumulation, despite similar food intake and body weight, when fed a methionine and choline deficient diet (Sinclair et al., 2008). We likewise found that female *Gcgr* ^−/−^ mice were prone to steatosis and dyslipidemia in response a GAN diet and HFD. In contrast, resistance to HFD‐induced steatosis has been reported in male *Gcgr* ^−/−^ mice (Conarello et al., 2007). We did observe that male *Gcgr* ^−/−^ mice were not as prone to steatosis when fed a HFD as female *Gcgr* ^−/−^ mice were, however, our study was not powered to test for sex differences. Taken together, these studies do not give a final and conclusive answer to how parameters of lipid metabolism are changed when glucagon receptor signaling is inhibited, however most data, including the data presented in the current paper, points to a change in hepatic lipid metabolism that may be reflected in elevated plasma cholesterol and perhaps TG concentrations. Also, by quantifying the liver TG content in *Gcgr* ^−/−^ mice fed a HFD and GAN diet enzymatically, we were unable to explain the histology results. This prompted us to perform lipidomics on liver biopsies from these mice and here we found significant differences in the lipidome based on genotype, suggesting that permanent deletion of the glucagon receptor has significant effects on hepatic lipid metabolism.

*Gcgr* ^−/−^ mice have been reported to have similar plasma insulin levels and similar pancreatic insulin content when compared to control mice (Gelling et al., 2009; Parker et al., 2002). This is consistent with *Gcgr* ^−/−^ mice showing improved glycemic control and increased insulin sensitivity (Gelling et al., 2009; Parker et al., 2002). Increased plasma levels of glucagon‐like peptide 1 (GLP‐1) and FGF‐21 were found to contribute to the improved glycemic control in *Gcgr* ^−/−^ mice and to work in a complementary way to prevent postprandial hyperglycemia in mice lacking insulin and glucagon action (Omar et al., 2014). Increased hepatic insulin sensitivity might contribute to the observed hepatic lipid and glycogen accumulation in *Gcgr* ^−/−^ mice. This is supported by one of our previous studies in which mice treated with a glucagon receptor antibody showed an upregulation of hepatic genes involved in insulin response (Galsgaard et al., 2022). Insulin is also a powerful regulator of adipocyte lipolysis; however, basal lipolysis was similar in white adipose tissue of *Gcgr* ^−/−^ and control mice while epinephrine stimulated white adipose tissue lipolysis was increased in *Gcgr* ^−/−^ mice when compared to control mice (Gelling et al., 2009). This might explain the observed tendency to increased free fatty acid plasma concentrations in *Gcgr* ^−/−^ mice.

It is evident from studies in *Gcgr* ^−/−^ mice that additional compensatory mechanisms exist to prevent hypoglycemia. These were investigated in (Gelling et al., 2009), suggesting that *Gcgr* ^−/−^ mice may compensate for loss of glucagon receptor signaling in part by increased basal cAMP signaling in the liver as well as an increased responsiveness to epinephrine in the liver and white adipose tissue as both basal and epinephrine stimulated cAMP levels in liver membranes from *Gcgr* ^−/−^ mice were increased and epinephrine stimulated (but not basal) white adipose tissue lipolysis was increased in knockout mice when compared to control.

Glucagon administration reduce food intake through reduced meal size and increased satiety (Bagger et al., 2015; Geary et al., 1992, 1993; Holloway & Stevenson, 1964; Langhans et al., 1982; Le Sauter & Geary, 1991; Martin & Novin, 1977; Penick & Hinkle Jr, 1961; Schulman et al., 1957; Stunkard et al., 1955; Weick & Ritter, 1986) and glucagon receptor agonism may result in body weight loss (Hinds et al., 2021; Pedersen et al., 2018). Conversely, treatment with certain inhibitors of glucagon receptor signaling have been reported to increase body weight (Engel et al., 2011; Guzman et al., 2017; Kazda et al., 2017). We found *Gcgr* ^−/−^ mice fed a standard chow diet to have increased body weight compared to *Gcgr* ^+/+^ mice, and this was associated with an increase in both lean and fat mass. In contrast to our findings, *Gcgr* ^−/−^ mice have been shown to have increased wholebody lean mass and decreased fat mass (Gelling et al., 2003; Hinds et al., 2021), and body weight have been found to be similar in whole body *Gcgr* ^−/−^ and wild‐type mice given a standard chow diet (Gelling et al., 2003). Mice carrying a naturally occurring deleterious glucagon receptor mutation also showed unaltered food intake and body weight with reduced adiposity (Lin et al., 2020). Mice with a partially inactivating mutation in the glucagon receptor gene showed increased body weight from 9 to 11 months of age (Xu et al., 2024). When fed a GAN diet and HFD, we found female *Gcgr* ^−/−^ mice to not gain weight whereas the *Gcgr* ^+/+^ mice gained weight as expected. This is consistent with a study showing that *Gcgr* ^−/−^ mice fed a HFD for 8 weeks did not gain weight while the wild‐type mice did (Longuet et al., 2008). Furthermore, wild‐type mice given a HFD for 12 weeks gained more weight than *Gcgr* ^−/−^ mice (Conarello et al., 2007). This was most likely due to the *Gcgr* ^−/−^ mice consuming less HFD than the wild‐type mice (Conarello et al., 2007). In both studies the *Gcgr* ^−/−^ mice had a reduced fat mass compared to wild‐type mice after the HFD feeding. However, in one of the studies (Longuet et al., 2008) the *Gcgr* ^−/−^ mice developed steatosis to a higher degree than the wild‐type mice, while in the other study the *Gcgr* ^−/−^ mice developed less steatosis compared to the wild‐type mice (Conarello et al., 2007).

The liver is a highly sexually dimorphic organ. In response to a short‐term fast, female mice use amino acids as substrates to increase lipid storage in the liver, whereas males simply slow down anabolic pathways (Della Torre et al., 2018). FGF‐21 regulates transcriptional and metabolic responses in the liver, and reduced liver TG concentrations, in a sex‐dependent manner (Chaffin et al., 2022). In diet‐induced obese male mice FGF‐21 decreases hepatic triglyceride levels (in a weight‐loss independent manner) and decreases body weight via loss of fat mass, neither of which were observed in diet‐induced obese female mice (Chaffin et al., 2022). FGF‐21 increased caloric intake and improved glucose tolerance in both male and female mice (Chaffin et al., 2022). FGF‐21 drives the reduction in liver TGs partly through adiponectin, whereas FGF‐21 did not increase adiponectin levels in female mice (Chaffin et al., 2022). FGF‐21 works via the brain to reduce adipose tissue, and it was suggested that FGF‐21 treatment (e.g., high FGF‐21 levels) promotes a “FGF‐21‐resistant” state in white adipose tissue of female mice thereby preserving lipid storage (Chaffin et al., 2022). These sex‐differences may to some degree explain the differences we observed in this study. We previously performed RNA sequencing of livers from *Gcgr* ^−/−^ and *Gcgr* ^+/+^ male mice fasted for 4 h and found FGF‐21 expression to be upregulated (Winther‐Sørensen et al., 2020), this is in line with the increased FGF‐21 plasma concentrations observed in both male and female *Gcgr* ^−/−^ mice. FGF‐21 has been reported to act centrally to induce sympathetic nerve activity, energy expenditure, and weight loss (Owen et al., 2014). Hypoglycemia also increases sympathetic nerve activity. Thus, it is possible that sympathetic nerve activity is increased in *Gcgr* ^−/−^ mice. This would potentially affect lipid metabolism and might explain the resistance to weight gain when *Gcgr* ^−/−^ mice were put on the GAN diet and HFD. However, an increased sympathetic nerve activity would contrast to the finding that the chow‐fed *Gcgr* ^−/−^ mice had increased body weight compared to *Gcgr* ^+/+^ mice and that *Gcgr* ^−/−^ mice had similar basal epinephrine levels compared to control mice (Gelling et al., 2009).

During fasting amino acids are released from the muscles, and catabolized and converted to glucose thus increasing ureagenesis (Li et al., 2016; Schimke, 1962); however, urea is rapidly excreted in the urine, a process that may be stimulated by glucagon (Ahloulay et al., 1995). Amino acid concentrations decreased during fasting in *Gcgr* ^−/−^ mice but remained unchanged in *Gcgr* ^+/+^ mice. The rapid urea excretion most likely explains the similar urea plasma concentrations in non‐fasted and fasted *Gcgr* ^+/+^ mice. The decreased urea index in *Gcgr* ^−/−^ mice compared to *Gcgr* ^+/+^ mice may reflect an impaired amino acid catabolism (decreased ureagenesis). The increase in the urea index upon fasting in *Gcgr* ^−/−^ mice may be caused by an increased urea plasma concentration due to impaired renal excretion (perhaps a consequence of glucagon receptor deletion as glucagon increase renal urea excretion (Ahloulay et al., 1992; Ahloulay et al., 1995) and the glucagon receptor is express in the kidney (Bomholt et al., 2021)), combined with a decreased amino acid plasma concentration due to lack of muscle derived plasma amino acids. In line with this urine urea in *Gcgr* ^−/−^ mice were reduced, however the daily excretion of urea in the urine and blood nitrogen levels were similar to wild‐type mice (Wang et al., 2024).

Glucagon‐based drugs are currently being investigated as therapeutic tools in the treatment of obesity and MASLD. In obese or overweight individuals with type 2 diabetes, a GLP‐1/glucagon co‐agonist decreased hepatic fat content (Robertson et al., 2020), and in mice with diet‐induced MASH the co‐agonist reduced body weight and liver TG concentrations (Trevaskis et al., 2020). A long‐acting glucagon analog decreased plasma and hepatic TG concentrations and decreased body weight in diet induced obese mice; the effect was mainly on fat mass and not lean mass (Lee et al., 2020). Glucagon analog treatment moreover resulted in a greater weight loss than GLP‐1 agonists (Lee et al., 2019). However a concern for loss of lean mass has been raised due to glucagon‐induced hypoaminoacidemia, this might be prevented by protein supplementation which can defend lean mass at a glucagon dose of that is sub‐anorectic and does not reduce fat mass, but still reduce steatosis and glycemia in obese mice (Hope et al., 2022; Lopes et al., 2024).

Our data show that female mice with genetic deletion of the glucagon receptor are prone to steatosis and dyslipidemia when fed a GAN diet and HFD. Thus, our data highlight glucagon as an important physiological regulator of not just glucose, but also hepatic lipid metabolism.

## AUTHOR CONTRIBUTIONS

Conceptualization; K.D.G., N.J.W.A., and J.J.H. Data curation; K.D.G., E.E., J.E.H., M.M.S., N.J.F., and J.H. Formal analysis; K.D.G., N.J.F., J.H., N.J.W.A., and J.J.H. Funding acquisition; K.D.G., N.J.W.A., and J.J.H. Project administration/supervision; N.J.W.A. and J.J.H. Writing—original draft; K.D.G. Writing—review and editing; E.E., J.E.H., M.M.S., T.J.G., C.C., N.J.F., J.H., N.J.W.A., and J.J.H. All authors approved the final version of the manuscript.

## FUNDING INFORMATION

The project is supported by grants from the A.P. Møller Foundation; NNF Tandem Programme (NNF Application Number: 31526); NNF Project Support in Endocrinology and Metabolism–Nordic Region (NNF Application Number: 34250). Katrine D. Galsgaard is supported by the BRIDGE‐Translational Excellence Programme (bridge.ku.dk) at Faculty of Health and Medical Sciences, University of Copenhagen, Copenhagen, funded by the Novo Nordisk Foundation. Grant agreement no. NNF20SA0064340. Associate Prof. Nicolai J. Wewer Albrechtsen is supported by NNF Excellence Emerging Investigator Grant – Endocrinology and Metabolism (Application No. NNF19OC0055001), EFSD Future Leader Award (NNF21SA0072746) and DFF Sapere Aude (1052‐00003B). Prof. Jens Juul Holst is supported by the Novo Nordisk Foundation (NNF) Center for Basic Metabolic Research University of Copenhagen (NNF Application Number: 13563). Novo Nordisk Foundation Center for Basic Metabolic Research is an independent Research Center, based at the University of Copenhagen, Denmark and partially funded by an unconditional donation from the Novo Nordisk Foundation (Grant number NNF18CC0034900 and NNF23SA0084103). Emilie Elmelund is supported by the Novo Scholarship Program (2022). N.J.F. received funding from Independent Research Fund Denmark (grant no. 2032‐00062B). Lipidomics work was performed within the Integra Infrastructure supported by the Novo Nordisk Foundation (NNF20OC0061575).

## CONFLICT OF INTEREST STATEMENT

None.

## PRIOR PRESENTATION

Some of the data were presented at American Diabetes Association's 82nd Scientific Session, June 3rd–7th, New Orleans, LA, USA.

## ETHICS STATEMENT

Animal studies were conducted at the animal facilities at the Faculty of Health and Medical Sciences, University of Copenhagen, Copenhagen, with permission from the Danish Animal Experiments Inspectorate, Ministry of Environment and Food of Denmark, permit 2018‐15‐0201‐01397. All studies were approved by the local ethical committee; Department of Experimental Medicine – University of Copenhagen.

## Supporting information


Appendix S1.

## Data Availability

All data reported in this paper will be shared by the lead contact upon request. All lipidomics data is available at Figshare (https://doi.org/10.6084/m9.figshare.27924528.v1).
