# Supplementary material for: Female glucagon receptor knockout mice are prone to steatosis but resistant to weight gain when fed a MASH‐promoting GAN diet and a high‐fat diet
Source: Physiol Rep. 2025 Feb 21;13(4):e70235. doi: 10.14814/phy2.70235 (PMC11845321; doi:10.14814/phy2.70235)
Supplement: Supplementary file 1 — Appendix S1. [file PHY2-13-e70235-s001.docx]

# **Supplementary data**


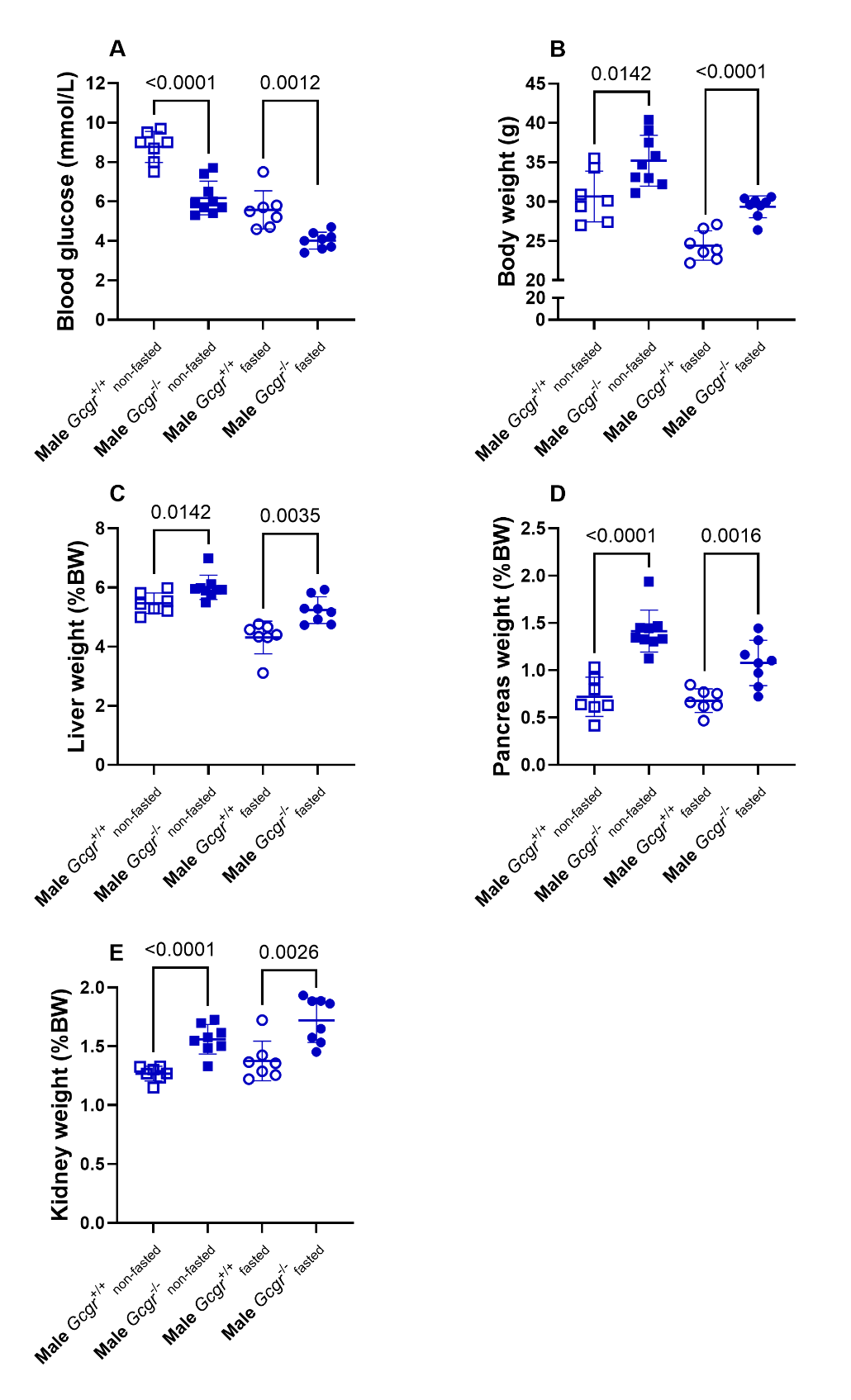


**Suppl. Fig. 1.** **Glucagon receptor knockout male mice show increased body weight.** (*A*) Blood glucose, (*B*) body weights, (*C*) liver weights, (*D*) pancreas weights, (*E*) kidney weights in non-fasted (squares) and overnight fasted (16 h) (circles) male wild-type littermates (*Gcgr*^+/+^, open symbols) and glucagon receptor knockout mice (*Gcgr*^-/-^, closed symbols). Data shown as mean±SD, n=7-8, mice 14-18 weeks of age. P-value by unpaired t-test.


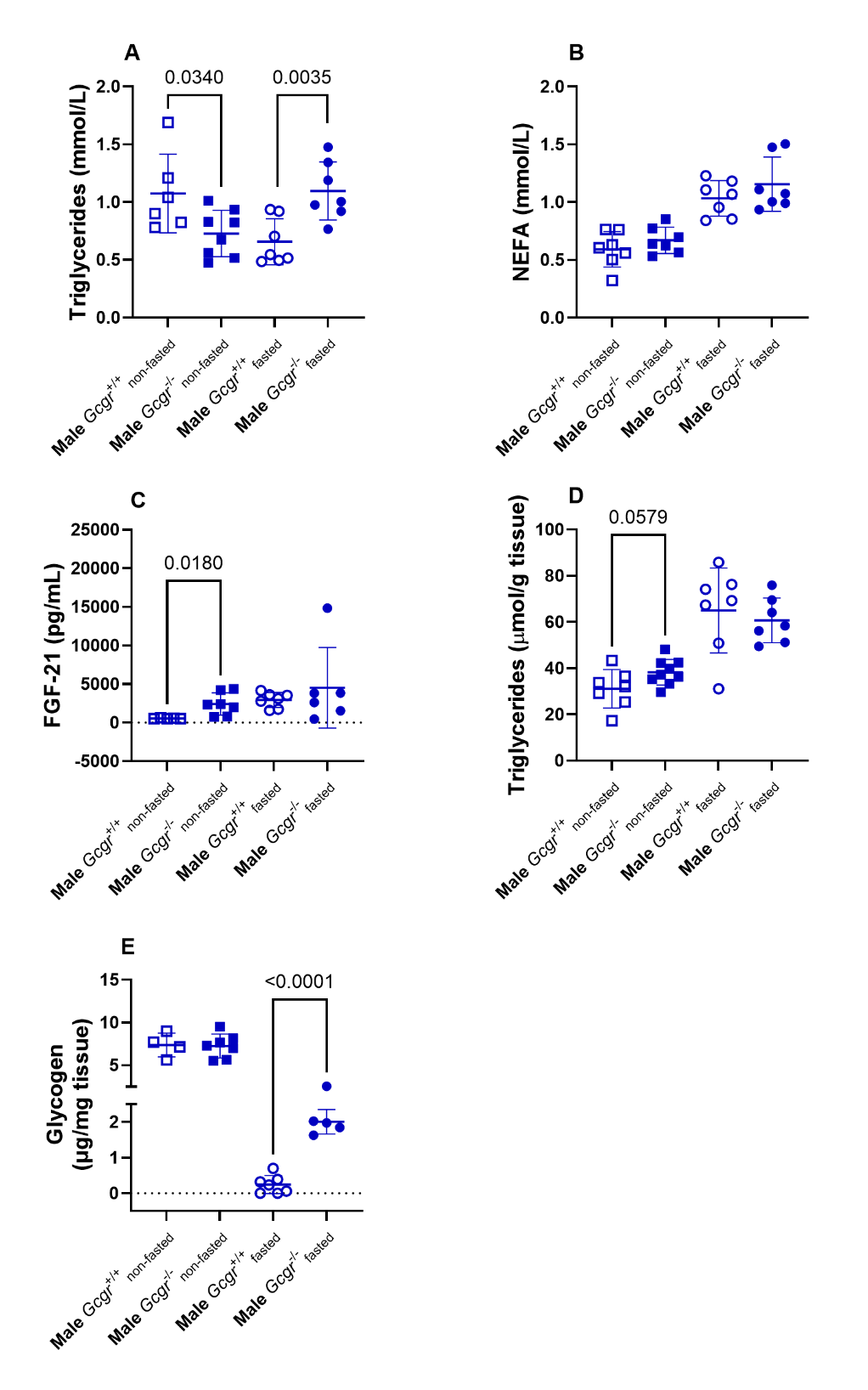


**Suppl. Fig. 2. Fasted glucagon receptor knockout male mice show hypertriglyceridemia.** (*A*) Plasma triglyceride, (*B*) non-esterified fatty acid (NEFA), (*C*) fibroblast growth factor 21 (FGF-21), (*D*) liver triglyceride, and (*E*) liver glycogen (2 of the measurements were under the detection limit and shown as 0 µg/mg) concentrations in non-fasted (squares) and overnight fasted (16 h) (circles) male wild-type littermates (*Gcgr*^+/+^, open symbols) and glucagon receptor knockout mice (*Gcgr*^-/-^, closed symbols). Data shown as mean±SD, n=4-9, mice 14-18 weeks of age. P-value by unpaired t-test.


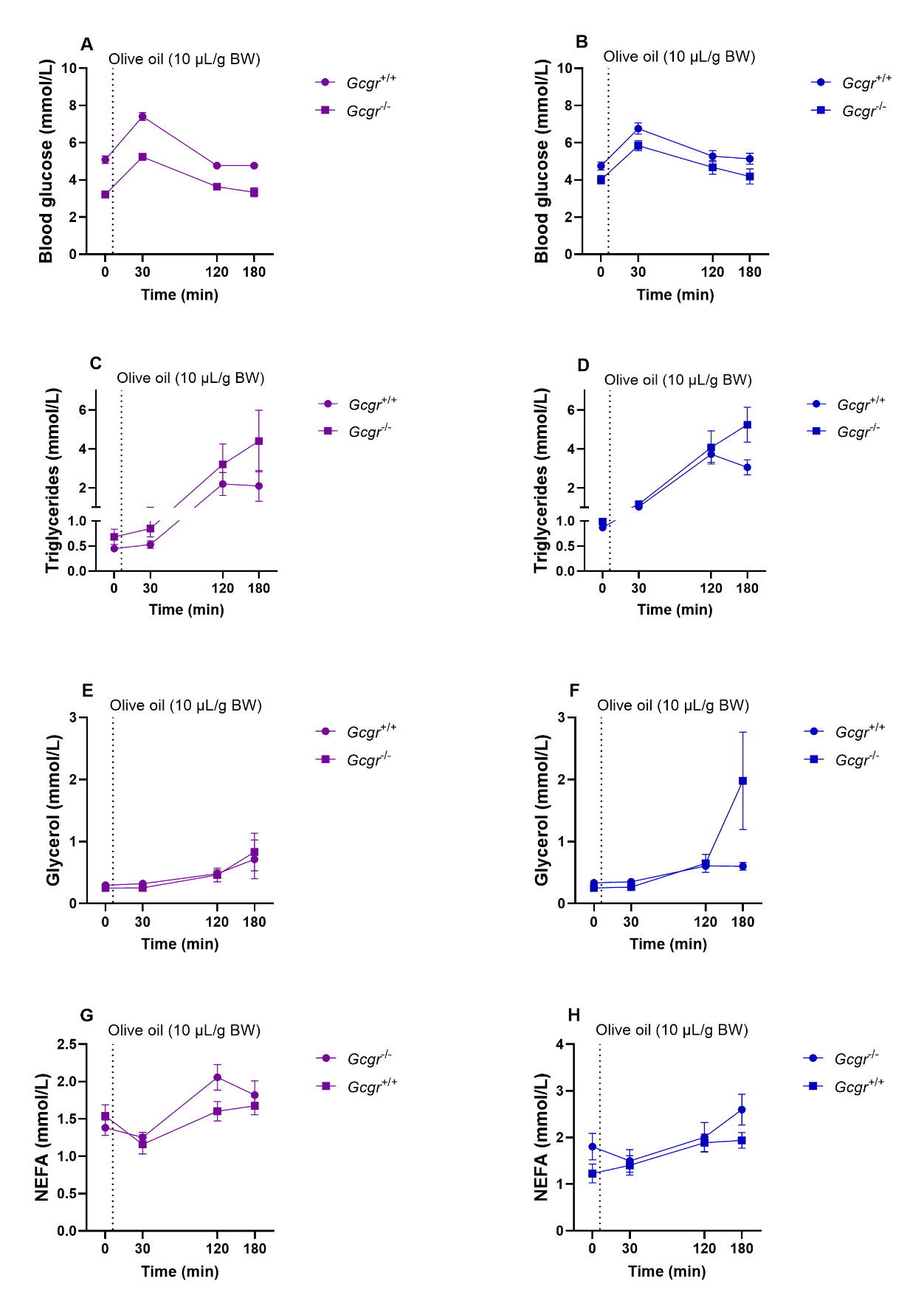


**Suppl. Fig. 3. Glucagon receptor knockout mice show lipid intolerance.** (*A, B*) Blood glucose, (*C, D*) plasma triglyceride, (*E, F*) glycerol, and (*G, H*) non-esterified fatty acid (NEFA) concentrations in glucagon receptor knockout mice (*Gcgr*^-/-^, circles) and their wild-type littermates (*Gcgr*^+/+^, squares) during a lipid tolerance test (olive oil, 10 µL/g body weight). Female mice shown in purple and male mice in blue. Data shown as mean±SEM, n=5-9, mice 10-14 weeks of age.


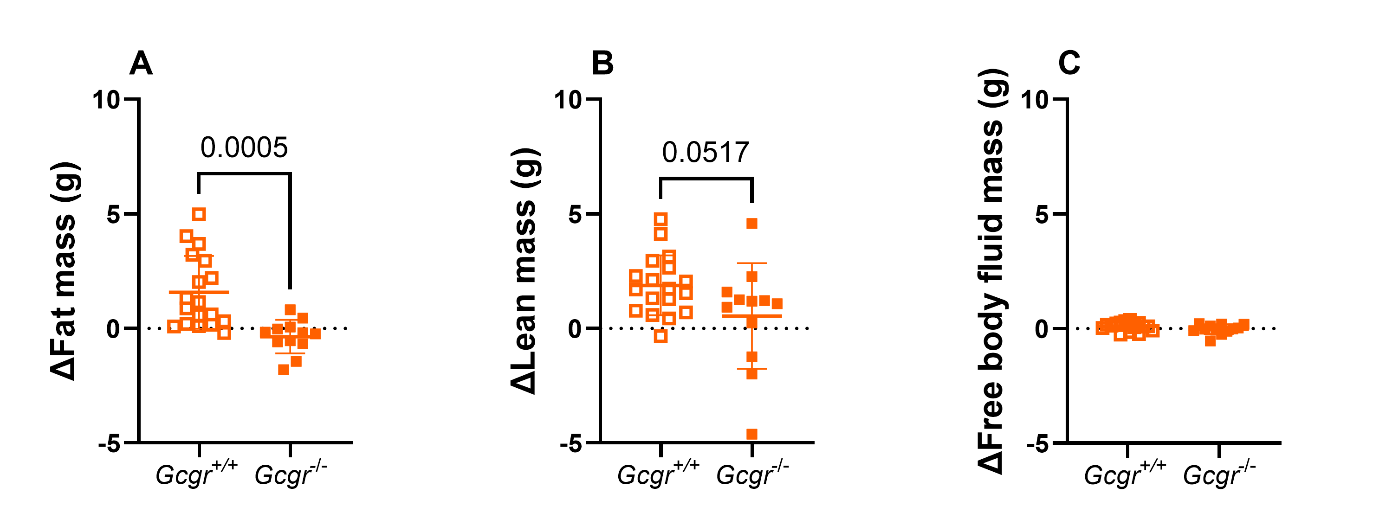


**Suppl. Fig. 4. Glucagon receptor knockout female mice gain less fat mass upon eight weeks HFD feeding.** (*A*) ΔFat mass (g), (*B*) Δlean mass (g), and (*C*) Δfree body fliud (g) in female wild-type littermates (*Gcgr*^+/+^, open symbols) and glucagon receptor knockout mice (*Gcgr*^-/-^, closed symbols) eight weeks after High Fat Diet (HFD) diet feeding. Data shown as mean±SD, n=12-18, mice 16-25 weeks of age. P-value by unpaired t-test.


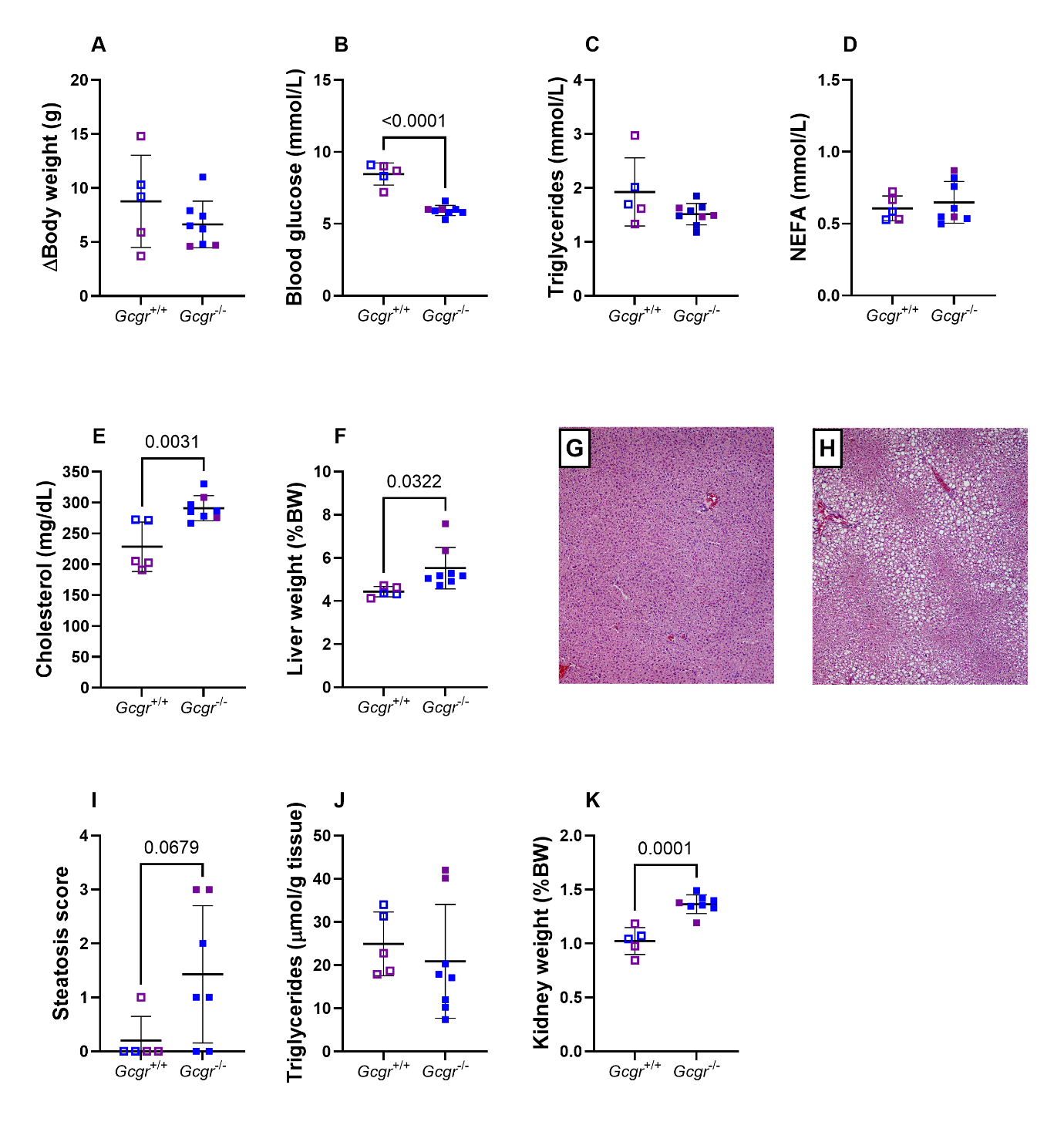


**Suppl. Fig. 5.** **Male glucagon receptor knockout mice may not be as prone to steatosis as female mice when challenged with a high fat diet.** (*A*) ΔBody weights, (*B*) blood glucose, (*C*) plasma triglyceride, (*D*) non-esterified free fatty acid (NEFA), (*E*) cholesterol, (*F*) liver weights, (*G*) representive H&E staining of female wild-type littermates (*Gcgr*^+/+^) and (*H*) glucagon receptor knockout mice (*Gcgr*^-/-^) livers, (*I*) steatosis score, (*J*) liver triglyceride concentrations, and (*K*) kidney weights in *Gcgr*^+/+^ (open symbols) and *Gcgr*^-/-^ (closed symbols) mice after eight weeks of High Fat Diet diet feeding. Values from male mice shown in blue and values from female mice shown in purple. Data shown as mean±SD, n=5-8, mice 13-16 weeks of age. P-value by unpaired t-test.


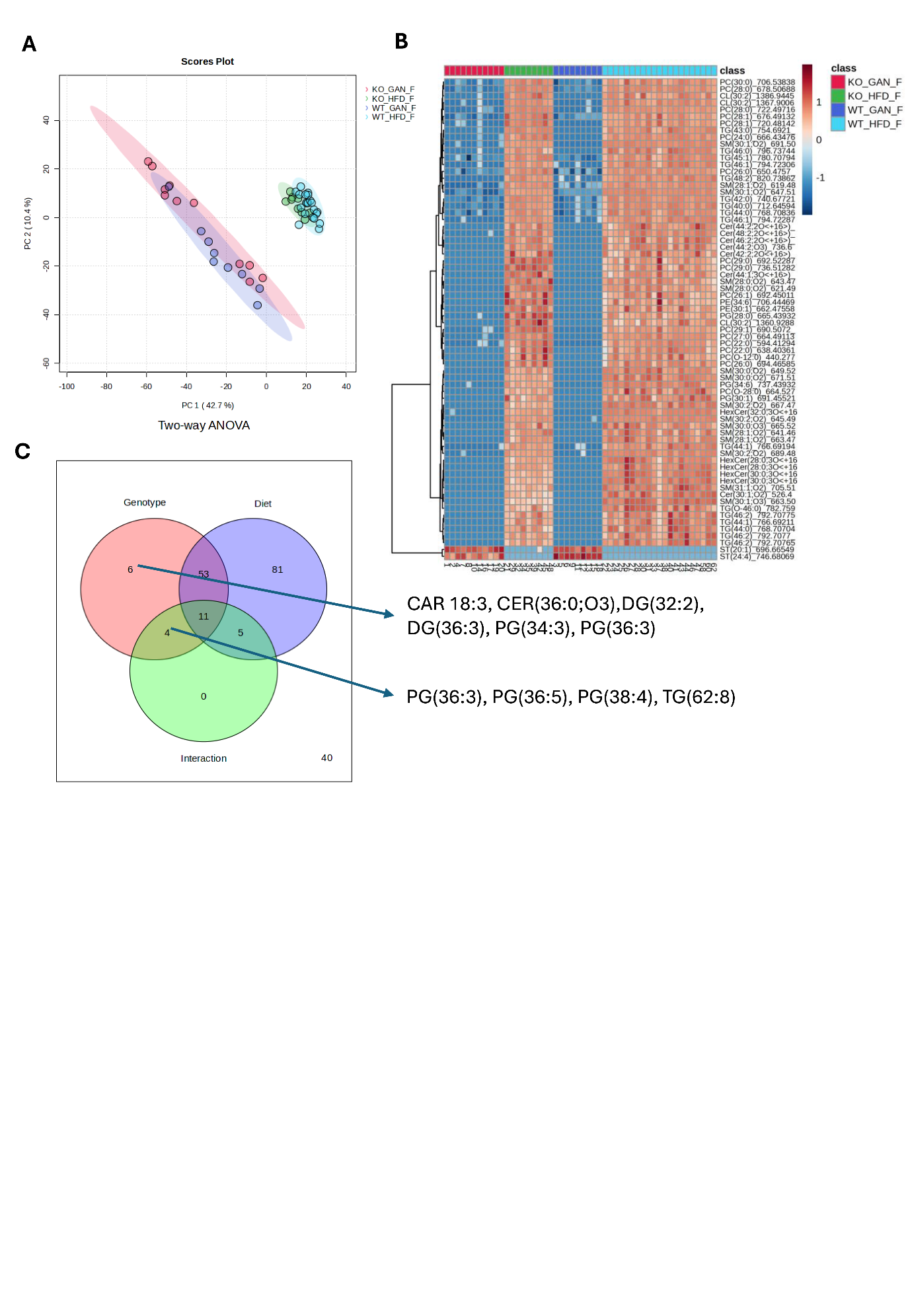


**Suppl. Fig. 6. Lipidomics analysis reveals significant difference in the lipidome of** **female mice challenged with a GAN and high fat diet.**  (*A*) Principal component analysis plot, (*B*) heat map and, (*C*) wenn diagram showing differencences in the lipodome of female glucagon receptor knockout mice (KO_GAN_F) and wild-type littermates (WT_GAN_F) after five weeks of Gubra Amylin Nonalcoholic (GAN) steatohepatitis diet feeding and female glucagon receptor knockout mice (KO_HFD_F) and wild-type littermates (WT_HFD_F) after eight weeks of high fat diet feeding.


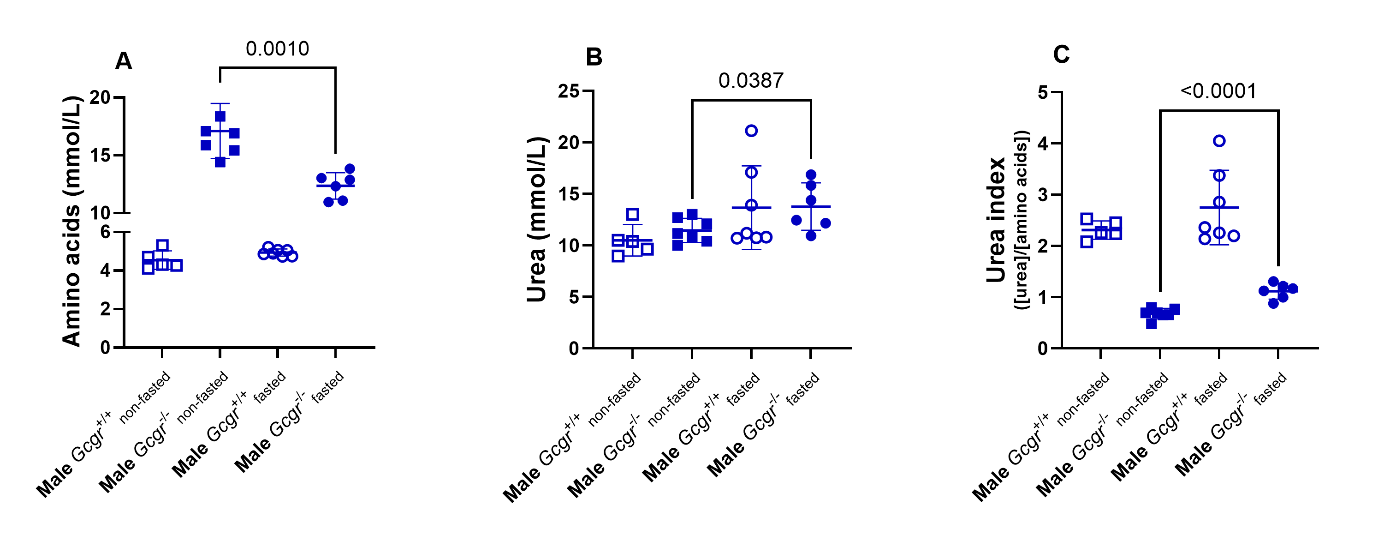


**Suppl. Fig. 7. Male glucagon receptor knockout mice show a decreased urea index.** (*A*) Plasma amino acid, (*B*) urea concentrations, and (*C*) urea index in non-fasted (squares) and overnight fasted (16 h) (circles) male wild-type littermates (*Gcgr*^+/+^, open symbols) and glucagon receptor knockout mice (*Gcgr*^-/-^, closed symbols). Data shown as mean±SD, n=5-7, mice 14-18 weeks of age. P-value by unpaired t-test.

**Suppl. Table 1. Top 20 up- and down-regulated lipid species in female glucagon receptor knockout mice challenged with a GAN diet.**

| ***Top 20 up-regulated*** | | | | |
| --- | --- | --- | --- | --- |
| **Name** | **FC** | **log2(FC)** | **raw.pval** | **-log10(p)** |
| PE(35:4)_726.50722_3.14_[M+H]+_MSMS | 8.5624 | 3.098 | 0.011337 | 1.9455 |
| DG(30:0)_558.50927_4.07_[M+NH4]+_MSMS | 5.6367 | 2.4949 | 0.0021029 | 2.6772 |
| LPC(17:0)_554.34593_1.72_[M+HCOO]-_MSMS | 5.1573 | 2.3666 | 0.046087 | 1.3364 |
| TG(71:2)_1143.09871_7.55_[M+NH4]+_MSMS | 4.7386 | 2.2445 | 0.0037589 | 2.4249 |
| SL 18:0;O/16:1;O_616.46149_2.6_[M-H]-_MSMS | 4.7358 | 2.2436 | 0.015104 | 1.8209 |
| Cer(39:3;2O<+16>)_896.73779_4.89_[M+HCOO]-_MSMS | 4.6863 | 2.2284 | 0.018502 | 1.7328 |
| DG(36:6)_630.5091_3.51_[M+NH4]+_MSMS | 4.2979 | 2.1036 | 0.033626 | 1.4733 |
| Cer(42:1;O3)_710.62957_4.74_[M+HCOO]-_MSMS | 4.2881 | 2.1004 | 0.049492 | 1.3055 |
| TG(69:1)_1117.08302_7.56_[M+NH4]+_MSMS | 4.2454 | 2.0859 | 0.0019223 | 2.7162 |
| DG(32:2)_582.50941_3.74_[M+NH4]+_MSMS | 3.9384 | 1.9776 | 0.01474 | 1.8315 |
| TG(70:1)_1131.09884_7.63_[M+NH4]+_MSMS | 3.9014 | 1.964 | 0.00676 | 2.1701 |
| DG(40:6)_686.57189_4.09_[M+NH4]+_MSMS | 3.8476 | 1.944 | 0.028047 | 1.5521 |
| PC(31:1)_762.52858_2.97_[M+HCOO]-_MSMS | 3.6063 | 1.8505 | 0.034766 | 1.4588 |
| TG(67:1)_1089.05171_7.44_[M+NH4]+_MSMS | 3.573 | 1.8371 | 0.0021029 | 2.6772 |
| DG(38:6)_658.54055_3.76_[M+NH4]+_MSMS | 3.5624 | 1.8328 | 0.014468 | 1.8396 |
| DG(37:3)_650.57174_4.35_[M+NH4]+_MSMS | 3.4896 | 1.8031 | 0.028859 | 1.5397 |
| TG(69:2)_1115.06773_7.42_[M+NH4]+_MSMS | 3.4742 | 1.7967 | 0.0065199 | 2.1858 |
| PC(31:0)_764.54436_3.32_[M+HCOO]-_MSMS | 3.4243 | 1.7758 | 0.029719 | 1.527 |
| PE(34:4)_712.49128_2.94_[M+H]+_MSMS | 3.2768 | 1.7123 | 0.029057 | 1.5368 |
| DG(36:4)_634.54062_3.87_[M+NH4]+_MSMS | 3.2668 | 1.7079 | 0.014109 | 1.8505 |
| ***Top 20 down-regulated*** | | | | |
| **Name** | **FC** | **log2(FC)** | **raw.pval** | **-log10(p)** |
| PG(38:6)_812.54405_2.62_[M+NH4]+_MSMS | 0.12316 | -3.0215 | 0.002073 | 2.6834 |
| TG(50:1)_850.78576_6.59_[M+NH4]+_MSMS | 0.15987 | -2.645 | 0.0021029 | 2.6772 |
| SM(34:1;O2)_1125.95071_6.2_[M+HCOO]-_MSMS | 0.16 | -2.6439 | 0.0014744 | 2.8314 |
| PC(36:2)_786.60085_4.1_[M+H]+_MSMS | 0.16602 | -2.5906 | 7.0816E-4 | 3.1499 |
| PC(38:4)_810.60055_4.63_[M+H]+_MSMS | 0.20101 | -2.3147 | 0.018604 | 1.7304 |
| SM(34:1;O2)_969.83534_5.54_[M+H]+_MSMS | 0.20289 | -2.3013 | 0.0065199 | 2.1858 |
| HBMP 18:1_18:1_18:0_1058.83519_5.4_[M+NH4]+_MSMS | 0.22507 | -2.1516 | 0.0044154 | 2.355 |
| PC(40:7)_832.58333_3.54_[M+H]+_MSMS | 0.2291 | -2.126 | 0.046087 | 1.3364 |
| CL 16:0_18:1_16:1_18:0_1403.99494_5.88_[M-H]-_MSMS | 0.25599 | -1.9658 | 0.0014744 | 2.8314 |
| PC(36:2)_786.60063_4.65_[M+H]+_MSMS | 0.25856 | -1.9514 | 0.033665 | 1.4728 |
| BMP(44:11)_886.55918_2.52_[M+NH4]+_MSMS | 0.25929 | -1.9474 | 0.0065199 | 2.1858 |
| SM(42:0;O3)_833.70993_4.33_[M+H]+_MSMS | 0.25936 | -1.947 | 0.0065199 | 2.1858 |
| SM(58:2;O3)_1097.91921_6.04_[M+HCOO]-_MSMS | 0.27591 | -1.8577 | 5.57E-5 | 4.2541 |
| BMP(44:12)_884.54369_2.37_[M+NH4]+_MSMS | 0.27862 | -1.8436 | 0.0099387 | 2.0027 |
| CAR 12:0_344.27964_0.83_[M+H]+_MSMS | 0.28019 | -1.8355 | 0.029719 | 1.527 |
| PC(O-40:4)_824.65235_4.16_[M+H]+_MSMS | 0.28123 | -1.8302 | 0.046087 | 1.3364 |
| PI(40:5)_930.60621_3.4_[M+NH4]+_MSMS | 0.29331 | -1.7695 | 0.01411 | 1.8505 |
| PC(O-44:7)_874.66799_4.13_[M+H]+_MSMS | 0.30585 | -1.7091 | 0.033129 | 1.4798 |
| SM(42:1;O2)_1123.93479_6.02_[M+HCOO]-_MSMS | 0.30787 | -1.6996 | 0.0014744 | 2.8314 |
| HBMP 18:1_18:1_16:0_1030.80299_5.16_[M+NH4]+_MSMS | 0.30995 | -1.6899 | 0.0081744 | 2.0875 |

**Suppl. Table 2. Top 20 up- and down-regulated lipid species in female glucagon receptor knockout mice challenged with a high fat diet.**

| ***Top20 up-regulated*** | | | | |
| --- | --- | --- | --- | --- |
| **Name** | **FC** | **log2(FC)** | **raw.pval** | **-log10(p)** |
| PG(36:5)_767.48643_2.65_[M-H]-_MSMS | 10.758 | 3.4274 | 1.2425E-5 | 4.9057 |
| PG(38:6)_812.54407_2.86_[M+NH4]+_MSMS | 9.2244 | 3.2055 | 1.4429E-5 | 4.8408 |
| PG(32:0)_721.50176_3.19_[M-H]-_MSMS | 8.3983 | 3.0701 | 3.8698E-5 | 4.4123 |
| LPG(16:0)_483.27249_1.31_[M-H]-_MSMS | 6.0108 | 2.5876 | 4.4729E-7 | 6.3494 |
| ST(18:1)_668.63365_6.72_[M+NH4]+_MSMS | 5.9439 | 2.5714 | 2.9049E-5 | 4.5369 |
| PG(38:5)_795.51727_2.89_[M-H]-_MSMS | 5.7534 | 2.5244 | 6.908E-7 | 6.1606 |
| PG(34:3)_762.52782_2.72_[M+NH4]+_MSMS | 5.437 | 2.4428 | 8.592E-4 | 3.0659 |
| DG(36:2)_638.57222_5.39_[M+NH4]+_MSMS | 5.4132 | 2.4365 | 9.3694E-4 | 3.0283 |
| PG(36:4)_769.50157_2.88_[M-H]-_MSMS | 5.3775 | 2.4269 | 4.4729E-7 | 6.3494 |
| PG(38:4)_816.57622_3.33_[M+NH4]+_MSMS | 5.2087 | 2.3809 | 8.1521E-7 | 6.0887 |
| PG(36:3)_771.51756_2.96_[M-H]-_MSMS | 4.7681 | 2.2534 | 1.3436E-6 | 5.8717 |
| PG(36:3)_790.55865_3.04_[M+NH4]+_MSMS | 4.7145 | 2.2371 | 1.053E-4 | 3.9776 |
| PG(34:3)_743.48618_2.67_[M-H]-_MSMS | 4.3781 | 2.1303 | 6.2386E-7 | 6.2049 |
| PG(30:0)_693.4708_2.84_[M-H]-_MSMS | 4.2946 | 2.1025 | 6.9691E-6 | 5.1568 |
| PG(32:1)_738.52866_2.94_[M+NH4]+_MSMS | 4.2898 | 2.1009 | 1.6966E-4 | 3.7704 |
| ST(24:1)_391.28502_1.07_[M-H]-_MSMS | 4.1634 | 2.0578 | 8.8126E-4 | 3.0549 |
| TG(54:4)_900.80169_6.2_[M+NH4]+_MSMS | 4.0442 | 2.0159 | 1.1992E-4 | 3.9211 |
| PG(32:2)_736.51272_2.68_[M+NH4]+_MSMS | 3.674 | 1.8773 | 4.9367E-4 | 3.3066 |
| PG(34:2)_745.5019_2.94_[M-H]-_MSMS | 3.3522 | 1.7451 | 4.4729E-7 | 6.3494 |
| PG(32:2)_717.47094_2.62_[M-H]-_MSMS | 3.3013 | 1.723 | 3.7556E-6 | 5.4253 |
| ***Top 20 down-regulated*** | | | | |
| **Name** | **FC** | **log2(FC)** | **raw.pval** | **-log10(p)** |
| SM(34:1;O2)_969.83534_5.54_[M+H]+_MSMS | 0.089344 | -3.4845 | 2.4233E-5 | 4.6156 |
| PI(38:2)_908.62227_3.78_[M+NH4]+_MSMS | 0.13204 | -2.921 | 5.0039E-5 | 4.3007 |
| PG(38:6)_793.50169_2.57_[M-H]-_MSMS | 0.19614 | -2.35 | 1.6096E-4 | 3.7933 |
| LPC(20:1)_550.38663_1.92_[M+H]+_MSMS | 0.23857 | -2.0675 | 0.0026758 | 2.5725 |
| PC(38:4)_854.59071_6.16_[M+HCOO]-_MSMS | 0.25551 | -1.9686 | 0.033709 | 1.4723 |
| SM(34:1;O2)_1125.95071_6.2_[M+HCOO]-_MSMS | 0.27255 | -1.8754 | 1.3436E-6 | 5.8717 |
| PC(36:2)_786.60124_5.37_[M+H]+_MSMS | 0.28429 | -1.8146 | 0.012572 | 1.9006 |
| SM(42:1;O2)_1123.93479_6.02_[M+HCOO]-_MSMS | 0.29472 | -1.7626 | 1.3436E-6 | 5.8717 |
| SM(58:2;O3)_1097.91921_6.04_[M+HCOO]-_MSMS | 0.30518 | -1.7123 | 1.4259E-5 | 4.8459 |
| PC(46:6)_962.68404_4.56_[M+HCOO]-_MSMS | 0.31414 | -1.6705 | 0.0026758 | 2.5725 |
| SM(54:2;O3)_1041.85648_5.68_[M+HCOO]-_MSMS | 0.31898 | -1.6485 | 2.5712E-6 | 5.5899 |
| Cer(39:2;O2)_650.57258_4.28_[M+HCOO]-_MSMS | 0.33352 | -1.5842 | 8.6129E-4 | 3.0649 |
| PE(36:0)_748.5852_4.48_[M+H]+_MSMS | 0.34196 | -1.5481 | 8.0624E-5 | 4.0935 |
| Cer(43:1;O2)_708.65038_5.22_[M+HCOO]-_MSMS | 0.35656 | -1.4878 | 0.0011946 | 2.9228 |
| PI(38:2)_889.58024_3.67_[M-H]-_MSMS | 0.37035 | -1.433 | 4.3912E-6 | 5.3574 |
| SM(52:2;O3)_1013.82576_5.47_[M+HCOO]-_MSMS | 0.37694 | -1.4076 | 7.1117E-7 | 6.148 |
| SM(40:1;O3)_847.65438_4.18_[M+HCOO]-_MSMS | 0.38017 | -1.3953 | 0.0012706 | 2.896 |
| CAR 12:0_344.27964_0.83_[M+H]+_MSMS | 0.38459 | -1.3786 | 2.2161E-5 | 4.6544 |
| SL 16:0;O/16:0;O_590.44581_2.39_[M-H]-_MSMS | 0.38885 | -1.3627 | 8.3317E-4 | 3.0793 |
| PC(44:12)_922.55938_2.64_[M+HCOO]-_MSMS | 0.39352 | -1.3455 | 0.0022108 | 2.6555 |
